# Cellulose-Based Nanofibers Infused with Biotherapeutics for Enhanced Wound-Healing Applications

**DOI:** 10.1021/acspolymersau.4c00092

**Published:** 2025-02-10

**Authors:** Deepanjan Datta, Sony Priyanka Bandi, Viola Colaco, Namdev Dhas, Suprio Shantanu Saha, Syed Zubair Hussain, Sudarshan Singh

**Affiliations:** † Department of Pharmaceutics, Manipal College of Pharmaceutical Sciences, Manipal Academy of Higher Education, Manipal, Karnataka State 576104, India; ‡ Department of Pharmacy, Birla Institute of Technology and Science (BITS) Pilani, Hyderabad Campus, Hyderabad, Telangana State 500078, India; § Department of Textile Engineering, Khulna University of Engineering and Technology, Khulna-9203, Khulna, Bangladesh; ∥ Faculty of Pharmacy, Chiang Mai University, Chiang Mai 50200, Thailand; ⊥ Office of Research Administrations, Chiang Mai University, Chiang Mai 50200, Thailand

**Keywords:** Nanofibers, Cellulose, Active therapeutics, Tissue Engineering, Biomaterials, Scaffolds, Dermal Infections, Wound Healing

## Abstract

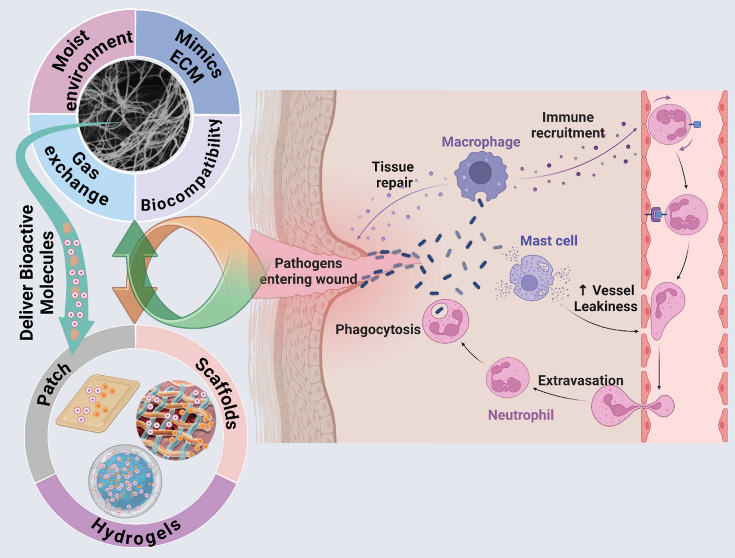

Nanofibers fabricated from various materials such as polymers, carbon, and semiconductors have been widely used for wound healing and tissue engineering applications due to their excellent nontoxic, biocompatible, and biodegradable properties. Nanofibers with a diameter in the nanometer range possess a larger surface area per unit mass permitting easier addition of surface functionalities and release of biotherapeutics incorporated compared with conventional polymeric microfibers. Henceforth, nanofibers are a choice for fabricating scaffolds for the management of wound healing. Nanofibrous scaffolds have emerged as a promising method for fabricating wound dressings since they mimic the fibrous dermal extracellular matrix milieu that offers structural support for wound healing and functional signals for guiding tissue regeneration. Cellulose-based nanofibers have gained significant attention among researchers in the fabrication of on-site biodegradable scaffolds fortified with biotherapeutics in the management of wound healing. Cellulose is a linear, stereoregular insoluble polymer built from repeated units of d-glucopyranose linked with 1,4-β glycoside bonds with a complex and multilevel supramolecular architecture. Cellulose is a choice and has been used by various researchers due to its solubility in many solvents and its capacity for self-assembly into nanofibers, facilitating the mimicry of the natural extracellular matrix fibrous architecture and promoting substantial water retention. It is also abundant and demonstrates low immunogenicity in humans due to its nonanimal origins. To this end, cellulose-based nanofibers have been studied for protein delivery, antibacterial activity, and biosensor applications, among others. Taken together, this review delves into an update on cellulose-based nanofibers fused with bioactive compounds that have not been explored considerably in the past few years.

## Introduction

**Figure 1:**
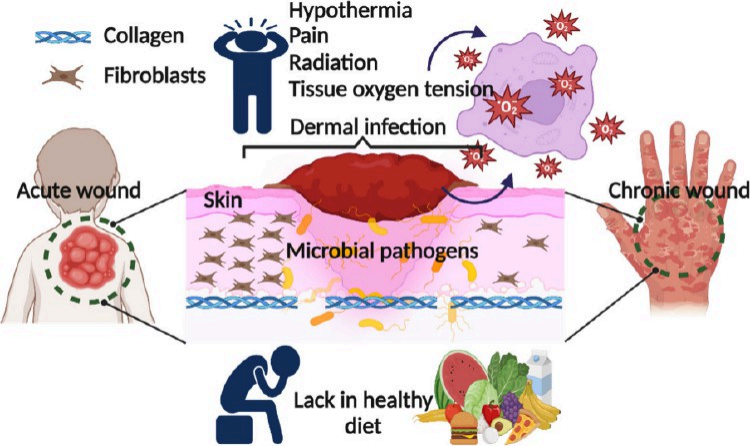
Schematic illustration of different local and systemic factors and their impact on the wound. This image has been created using the Creative Common Biorender software (https://www.biorender.com/).

Skin, the largest organ in the human body, has a considerable impact on various human activities and functions, such as pathogen defense, environmental sensing, and temperature regulation. The epidermis and dermis are two layers that make up its structure.^1−3^ The skin’s essential barrier is predominantly located in the uppermost layer of the epidermis, the stratum corneum (SC). The epidermis is divided into multiple layers, beginning with the base layer or stratum basale, situated above the dermis, and progressing via the prickle and granular layers to the outermost SC layer.^4^ The epidermis comprises the epidermal–dermal basement membrane, keratinocytes, melanocytes, dendritic cells, Langerhans cells, and other immune cells. The dermis layer consists of skin appendages, mast cells, fibroblasts, antigen-presenting dermal cells, and resident and circulating immune cells.^5^ Moreover, the dermis also contains the extracellular matrix complex, which controls the actions of cytokines and growth factors, and supports intercellular connections and cellular mobility.^6^ However, due to the elastic and delicate nature of the skin, it is exposed to constant conflict, susceptible to producing deformities known as wounds. In other words, “wounds” can be defined as disruptions in the continuity of the epithelial lining of the skin or mucosa resulting from thermal or physical injury. Even though human skin can spontaneously self-heal to restore its structural and functional integrity, wound care is still vital to avoid scars, infections, and desiccation, relieving pain, protecting the open wound site, and accelerating healing. This is especially true for large or open wounds and burns.^7−9^ Wounds can be classified as acute and chronic, based on the duration and nature of the healing process.^10,11^ An injury to the skin caused by an accident or surgical procedure is termed an acute wound. Depending on the size, depth, and degree of the injury to the epidermis and dermis layers of the skin, it normally recovers within 8 to 12 weeks.^12,13^ On the other hand, chronic wounds cannot be cured in a timely and ordered fashion. This can only be determined retrospectively, which is clinically invaluable. Burns, leg ulcers, and decubitus ulcers, among others, are the most common causes of chronic wounds. In addition, wounds can also be evaluated based on the wound characteristics including slough, exudate, maceration, and infections, followed by host factors such as critical limb ischemia and host immune responses.^14,15^ The factors affecting the wound healing process have been broadly discussed throughout the history of wound care. Local factors including cold, pain, infection, radiation, and tissue oxygen tension have a direct impact on the wound, whereas systemic factors are related to an individual’s overall health or disease condition. These factors influence the individual’s ability to heal progress. Along with these factors, a lack of protein, vitamins, minerals, and advancing age might further delay the healing process (Figure 1).

**Figure 2:**
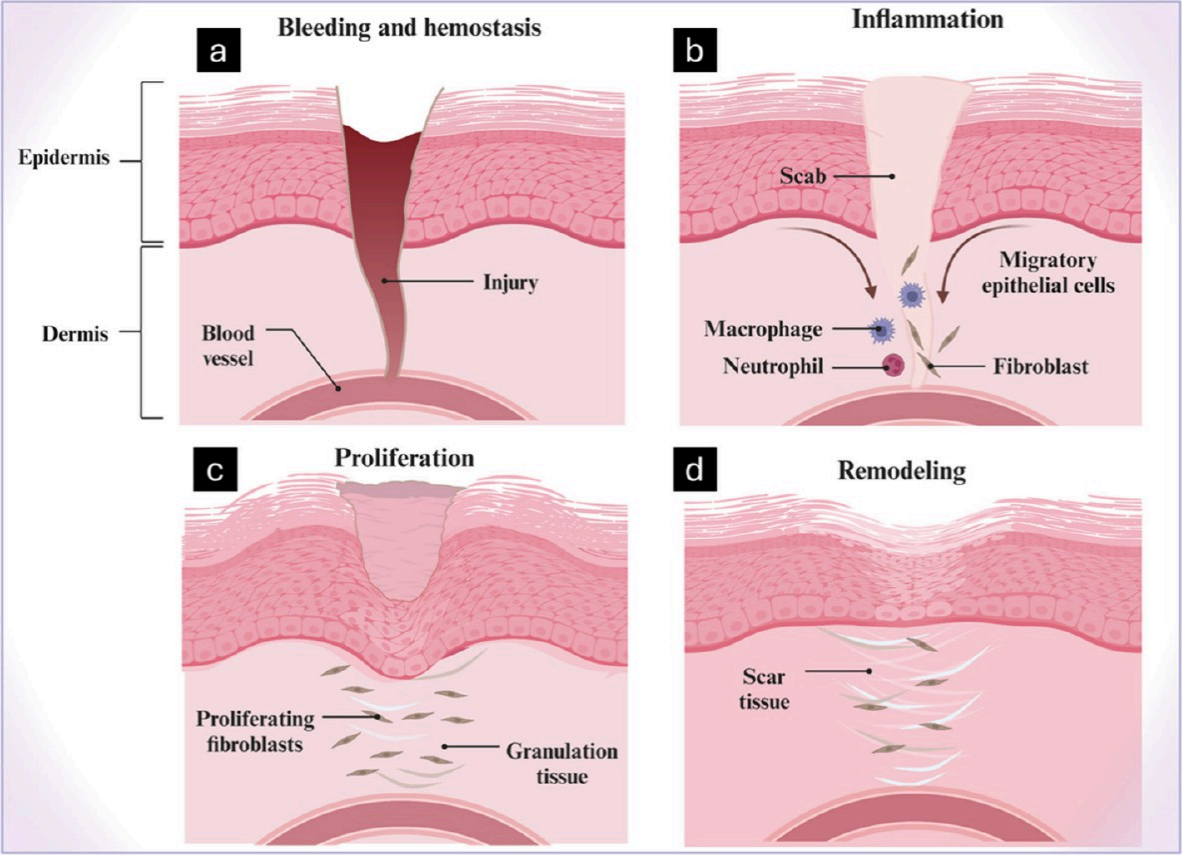
Figure depicts several phases of wound healing: Neutrophils invading the wound area (a). Epithelial cells infiltrate the wound region (b). Full epithelial covering of the wound (c). Dissipation of the capillaries and fibroblasts that developed during the earliest phases (d). This image has been created using the Creative Common Biorender software (https://www.biorender.com/).

The process of wound healing is intricate, relying on a multitude of cellular activities that must be closely synchronized to effectively restore the injured tissue. To date, there are four interconnected stages in the healing process which are hemostasis; inflammation; proliferation; and remodeling (Figure 2). Several growth factors, enzymes, cytokines, and other molecules work together and have a major influence in synergistically altering cell activities.^16^ The first stage of hemostasis involves the formation of an insoluble eschar of fibrin, fibronectin, vitronectin, and thrombospondin, which primarily plug and prevent wound bleeding. The second stage of inflammation is considered as the primary defense against pathogenic invasion. Damage-associated molecular patterns (DAMPs) and pathogen-associated molecular patterns (PAMPs) activate resident immune cells by binding to the receptors to provoke downstream inflammatory pathways.^17^ The release of selectins as an important pro-inflammatory molecule has been shown to facilitate neutrophil, monocyte adhesion, and diapedesis.^18^ These have been shown to cause the blockade of E -and P-selectin significantly, responsible for the impairment of cell filtration and wound healing.^19,20^ In the third stage, the proliferative phases of healing, wound closure, matrix deposition, and angiogenesis are orchestrated by activated keratinocytes, fibroblasts, macrophages, and endothelial cells. The fourth stage involves the remodeling of the extracellular matrix which initially begins with the deposition of fibrin clots and ends with the formation of mature, type-I collagen-rich fibrils, which increases the tensile strength of the wounded scar.^21^ For most wounds, the extracellular matrix (ECM) architecture fails to return to that of the original unwounded skin. However, for superficial injuries or wounds, the full regeneration of the ECM architecture is anticipated based on the coordinated actions of various cells and enzymes that include matrix metalloproteinases, fibroblasts, macrophages (growth factors like VEGF and TGF-β), integrins and angiogenesis, among others.^22,23^

Skin regeneration is the most complex biological and dynamic process that involves the constant interplay of various peptide growth and hormonal factors with receptors for the mediation of re-epithelization. Hepatocyte growth factor, epidermal growth factor, transforming growth factor, and fibroblast growth factor among others get bonded to tyrosine kinase, threonine kinase, G-protein coupled receptor and nuclear receptor for the stimulation or inhibition of keratinocyte migration or proliferation and survival.^24^

The therapeutic approaches for wound treatment are diverse, encompassing many methods such as hyperbaric oxygen therapy,^25^ negative-pressure therapy,^26^ vacuum-assisted closure,^27^ ultrasound,^28^ electrotherapy,^29^ auto/allograft and xenograft,^30^ cell-based therapy,^31^ and engineered skin graft,^32^ as well as topical drug and growth factor delivery.^33^ In addition, the application of biomaterials has shown positive implications on wound repairment due to their intrinsic features such as biocompatibility, the absence of cytotoxicity and antigenic or inflammatory stimulation, and a compatible biodegradability with the rate of formation of new tissues.^34^ They have been shown to prevent infections, and the ability to fight against microbial pathogens that colonize wounds, when treated, modified or loaded with active agents or therapeutic agents.^35−39^ The porous nature of the biomaterials also ensures the permeability of water and gases, as well as the capacity to retain moisture at the wound site, to expedite and enhance the efficacy of wound healing.^40^ To this end, various synthetic and natural-based biopolymers have been used in wound healing.^41^ These biopolymers were used for the fabrication of semipermeable films, foams, hydroactive dressings, hydrocolloids, nanofibers, nanogel, emulgels and bigels, among others, which significantly enhance wound healing.^42−46^ Among all of these, nanofibers have enhanced the scope of developing scaffolds that can mimic the architecture of the tissues in the nanoscale. Polymers are processed in a certain way to create nanofibers, which are fine threads with diameters ranging from a few micrometers to nanometers. Nanofibers are the optimal matrix for developing extremely fine structures due to their high surface-to-volume ratio and their amenability to the alteration of surface characteristics. Immobilization or encapsulation of antibiotics, enzymes, antimicrobial peptides, and growth hormones into fiber matrices has shown opportunities in the progression of wound healing. Taken together, we understand that the management of wound healing is difficult, and thereby, it is still a challenge that attracts great interest among the researchers.

## 2 Tissue-Engineered Dermal Substitutes

The primary function of the skin is to provide a protective barrier and shield the body from dangerous substances and wounds. The layers of the skin are composed of ECM which is made up of collagens and glycosaminoglycans (GAGs) as a scaffold, in addition to keratinocytes, fibroblasts and functional cells.^47^ It is understood that for injured or wounded skin these components are compromised. Large surface area wounds render natural wound healing ineffective. Scientists in the field of tissue engineering have been shown to create artificial constructs that imitate the natural healing process to facilitate skin repair in untreatable wounds. Tissue engineering is a multidisciplinary field of research that is carried out all over the world due to the possibility of obtaining living tissue replacements and thus minimizing the dependency on donor tissue and organs. Recent years have witnessed significant advancements in the development and clinical application of bioengineered skin substitutes (BSS).^48,49^ Skin substitutes were fabricated as a successful treatment to lessen the risks associated with traditional skin transplantation. Skin substitutes must fulfill the requirements as mentioned below:

(a) Skin substitutes should be protective with supportive and mechanical strength to bear mechanical tensions and sheer forces.^50^
(b) Skin substitutes should provide a suitable surface for adherence and biological functions to provide an ideal environment for cell proliferation and differentiation.^51^ Their cell differentiation is ensured by the rapid adhesion of the skin substitutes to the wound surface.^48^
(c) The developed skin substitutes should have minimal adverse effects without causing any inflammatory response of the immune system. Studies have shown that skin substitutes are more suitable for clinical applications due to their absence of antigenicity, toxicity, immunogenicity, and low risk of disease transmission.^48,52^
(d) Patient compliance, where these skin substitutes should be easy to handle and comfortable to apply even on irregular wound surfaces such as knees, hips, and hands.^53,54^
(e) Skin substitutes should be stable and biodegradable as they are intended to replace the patient’s skin. Apart from interfering with the dermis vascularization, they should also maintain their structure until complete vascularization which usually takes a week time.^48,53,54^
(f) Skin substitutes should be developed considering the long-term storage and cost-effectiveness with long shelf life.^48,53,54^

**Figure 3:**
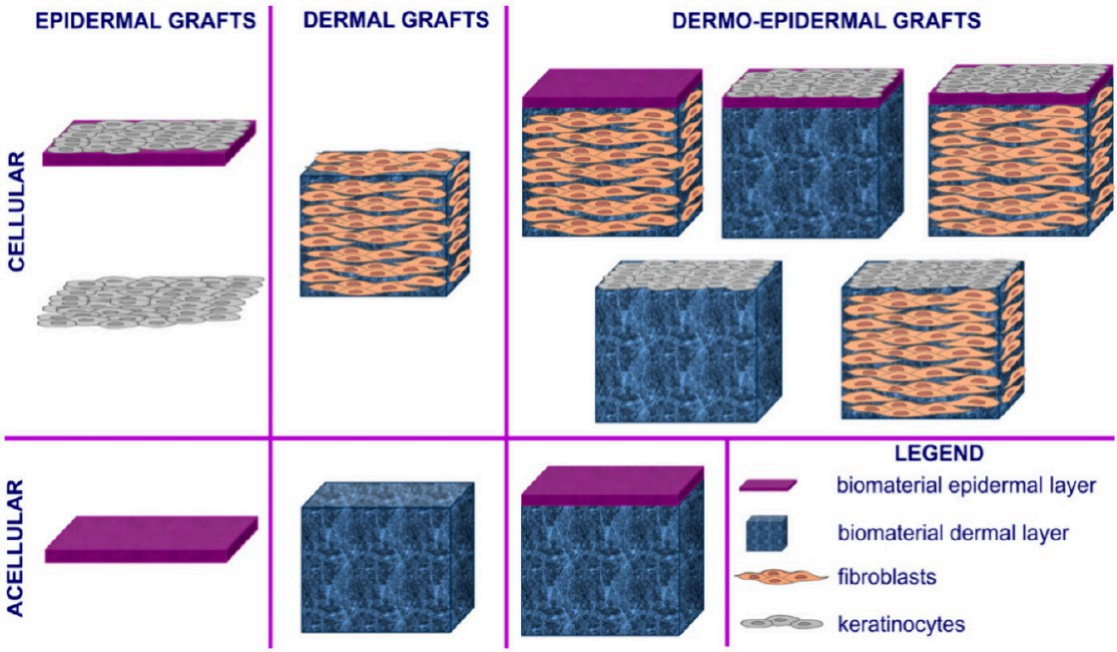
Diagram illustrating different types of tissue-engineered skin substitutes. Reproduced from ref (57). This is an open-access article, available under the terms of the Creative Common CC-BY license. Copyright 2020, Agata Przekora.

Epidermal, dermal, and dermo-epidermal are the three primary categories of commercially accessible engineered skin substitutes.^55^ Each of them might include growth factors, target cells, or scaffolds. Allogeneic, xenogeneic, or autologous origins are all possible for these replacements.^56^ Additionally, they could be cellular or acellular.^52^ These are used to relieve pain by speeding wound healing and restoring normal skin processes. The different types of skin substitutes are discussed below which have shown to mimic the ECM properties of the natural skin and their effectiveness in the management of wound healing (Figure 3).

### 2.1 Epidermal Substitutes

**Figure 4:**
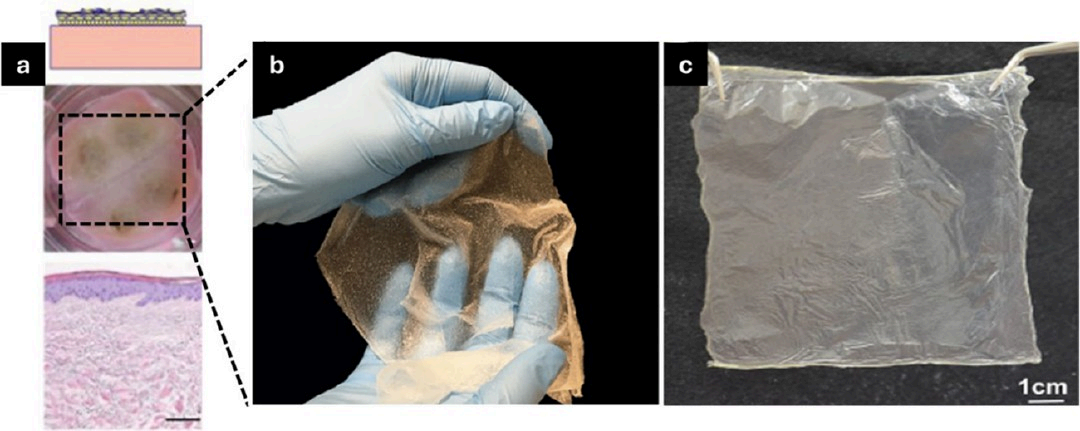
Upper and lower panels show the macroscopic image and hematoxylin and eosin staining of the tissue without dermal fibroblasts, respectively (a). Image representing epidermal substitute cultured on a chitosan/agarose nanofibrous membrane (b). Image representing the fabrication of cryopreserved human dried allogenic cultured epidermis (CE) (c). Reproduced from ref (59). This is an open-access article available under the terms of the Creative Common CC-BY license. Copyright 2019, Hanneke N. Monsuur, Ester M. Weijers, Susan Gibbs, Lenie J. van den; Reproduced from ref (60). This is an open-access article available under the terms of the Creative Common CC-BY license. Copyright 2022, Michiharu Sakamoto, Takashi Nakano, Itaru Tsuge, Hiroki Yamanaka, Yasuhiro Katayama, Yoshihiro Shimizu, Yoshika Note, Masukazu Inoie, Naoki Morimoto; Reproduced from ref (61). This is an open-access article available under the terms of the Creative Common CC-BY license. Copyright 2020, Vladyslav Vivcharenko, Michal Wojcik, and Agata Przekora.

Epidermal substitutes were initially prepared from cultured keratinocytes as stratified cell sheets. In this process, a skin biopsy of 2 to 5 cm^2^ is needed to provide the autologous keratinocytes that last for 3 weeks as epidermal replacements.^50^ Epidermis and dermis are separated following biopsy, and keratinocytes are then enzymatically extracted and cultivated *in vitro*.^58^ The result of using autologous keratinocytes is a culture epithelial autograft (CEA). Figure 4 shows the development of various CEA-based epidermal substitutes reported for wound treatment.

### 2.2 Dermal Substitutes

Dermal substitutes must only be applied to clean and well-prepared wounds. After 3–4 weeks of the transplant, the patient’s skin cells beneath the substitute get colonized and vascularized forming an autologous neo-dermis.^62^ This additional layer facilitates the detachment of the silicone cover, which can be substituted with a split-thickness skin graft (STSG) through an alternative surgical procedure.^63^ The wounds treated with these types of substitutes have shown better mechanical stability and less contraction when compared to CEA.^58^ To this end, collagen-GAG scaffold is available clinically as a first dermal substitute for wound treatment.^50^ Despite recent advancements in the development of dermal substitutes, these methods do not appear to be an optimal strategy. The development and utilization of dermal replacements not only require extensive surgical procedures accompanied by pain and problems but are also expensive. To this end, the combination of both dermal and epidermal substituents has been shown to yield superior results, particularly in cases involving full skin-thickness damage.

### 2.3 Combination of Epidermal and Dermal or Dermo-Epidermal Substitutes

**Figure 5:**
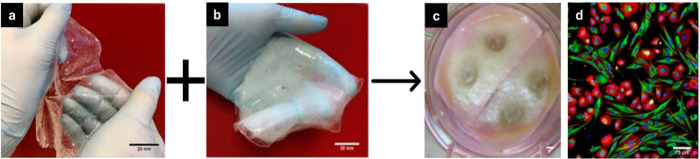
A combination of epidermal and dermal skin grafts forms dermo-epidermal skin composites. Epidermal skin graft of chitosan/agarose film (a) and dermal skin graft of chitosan/Curdlan film (b). The combination of human skin fibroblasts (dermal) and keratinocytes (epidermal) results in the formation of dermo-epidermal substitutes (c). Confocal microscopic images were captured for composite preparation, where blue and red fluorescence indicated nuclei and actin filaments of fibroblasts and keratinocytes, respectively. Green fluorescence indicates vimentin filaments of fibroblasts (d). Reproduced from ref (69). This is an open-access article available under the terms of the Creative Common CC-BY license. Copyright 2020, Agata Przekora; Reproduced from ref (70). This is an open-access article available under the terms of the Creative Common CC-BY license. Copyright 2019, Hanneke N. Monsuur, Ester M. Weijers, Susan Gibbs, Lenie J. van den.

Dermo-epidermal substitutes represent the most intricate and costly skin substitutes currently accessible in clinical settings. They function as substitutes for both the dermal and epidermal layers of typical skin.^64^ The first reported tissue-engineered dermo-epidermal substitute was conducted in the year 1981, utilizing allogeneic human cells.^50^ These bilayered skin analogues consist of autologous or allogeneic keratinocytes and fibroblasts that have been seeded onto 3D scaffolds.^49,65^ Autologous cells are derived from a biopsy of the damage site and subsequently incorporated into a construct based on collagen-glycosaminoglycan (GAG). Subsequently, the substitute can be transplanted following a cultivation period of 4 weeks.^66,67^ Dermoepidermal skin substitutes offer advantages over dermal or epidermal substitutes due to their advanced structure and function, providing a suitable skin barrier, nonimmunogenicity, release of growth factors/cytokines, and ECM deposition, among others.^58,68^ TiscoverTM (A-Skin) and StrataGraftTM are the commercially available dermo-epidermal skin substitutes developed by Advanced Tissue Medicinal Product, Netherlands and Striates Corporation, USA, respectively.^52^Figure 5 shows the development of co-cultured skin substitutes from dermal and epidermal grafts.

Polymeric nanofibrous scaffolds interact with and control particular regenerative processes at the molecular level to repair damaged tissues. So far it is understood that tissue-engineered biomaterial (skin substitutes) or scaffolds are required to provide covering and to promote tissue regeneration for the dermal wounds with excessive skin loss. These scaffolds should shield the wound bed from fluid loss, aid in exudate drainage, and protect against infection.^71^ Studies have shown that scaffolds made up of micro or nano polymeric fibers can mimic the inherent topography of the extracellular matrix of the skin, which offers greater advantages compared to skin grafts or substitutes.^72−75^ Gels, nano- and microfibers, films, and membranes have been considered as dermal replacement scaffolds. Among all these, nanofibers are the promising candidates due to (i) mimicking the native ECM; (ii) comparable mechanical properties to that of the skin; (iii) high porosity; (iv) high surface area-to-volume ratio; (v) enabling oxygen permeation; (vi) restricts the invasion of microorganisms; (vii) enhances the visual appearance of the repaired tissue; (viii) promotes cell adhesion and (ix) surface modification among others.^76^

Extensive research has been conducted on the utilization of electrospun nanofibers in the field of skin tissue engineering. A diverse array of polymers has been subjected to the electrospinning process and subsequently assessed for their capacity to facilitate the regeneration of skin tissue. To this end, various polymeric nanofibrous scaffolds were investigated including cellulose, gelatin, collagen, silk fibroin, poly(lactic-*co*-glycolide), polycaprolactone, grafted chitosan, hyaluronic acid, and polyurethane, among others (Table 1).

**Table 1:** Summary of the Polymeric Nanofibrous Scaffolds in Skin Tissue Engineering

Nanofibrous polymeric material	Advantages	Diameter size (nm)	Study type	References
Cellulose	Resembles skin’s ECM with high porosity and permeability, improved mechanical, and antibacterial properties	350–650	*In vitro* cell culture studies using L929 murine fibroblastic cell lines and animal models for healing diabetic foot ulcer	(77,78)
Collagen	Resembles skin’s ECM	190–430	The full-thickness wound was implanted in athymic mice.	(79−81)
Gelatin	Facilitates cell adhesion is facilitated and aids in exudates removal	70–170	Human skin fibroblasts in *in vitro* studies	(80,82,83)
Silk fibroin	Exhibits cytocompatibility with human keratinocytes and fibroblasts	12–1500	Human skin keratinocytes and fibroblasts in *in vitro* studies	(84,85)
poly(lactic-*co*-glycolide) (PLGA)	Possess antiadhesive properties	150–225	Human skin keratinocytes in *in vitro* studies	(71)
Polycaprolactone (PCL)	Enhanced cell infiltration	347–400	Human keratinocytes in *in vitro* culture studies	(86,87)
Chitosan	Elimination of exudates and supports the process of wound healing within a time frame of 14 days	100–300	Applied in patients with third-degree burn	(80,88−90)
Polyurethane	Favorable mechanical strength and promotes a moist environment	170–260	*In vitro* studies cultivating 3T3 mouse fibroblast cell lines	(91−94)
Hyaluronic acid	Natural occurrence in ECM with exceptional biocompatibility	244–326	*In vitro* studies using HaCaT and primary normal human dermal fibroblasts (NHDF) cell lines, proliferating keratinocytes, and fibroblasts	(80,95−97)
Poly(l-lactide) (PLLA)	Exceptional biocompatibility and mechanical strength	656–860	*In vitro* studies using L929 fibroblast cell lines for the evaluation of cell attachment and adhesion	(98−100)

## 3 Fabrication Technique of Nanofibers

Several fabrication methods have been employed to fabricate nanofibers since they were started four centuries ago. During the 1930s, Anton Formhals obtained the initial of multiple patents that disclosed the apparatus and technique for utilizing electrical energy to spin synthetic fibers from cellulose acetate solutions.^101,102^ Research interest intensified in the late 1990s, leading to more rapid industrial adoption of electrospinning in the subsequent decade due to swift technological advancements.^103−105^ The electrospinning technology enabled the production of fibers in various forms, including yarn, membranes, and 3D scaffolds.^106−108^ Nowadays, more than 300 academic institutions and research work centers are studying different methods of nanofiber fabrication and analyzing its substantial physical, mechanical, chemical, and other characteristics.^109^ Broadly, the nanofiber fabrication method can be classified under two main categories i.e., top-down approach and bottom-up approach.^110^

### 3.1 Top-Down Approaches

The top-down strategy entails the isolation and extraction of cellulose nanofibrils (CNFs) and nanocrystals (CNCs) from diverse natural resources, with the dimensions of the isolates constrained by the cellulose source and extraction methods employed. This approach results in the breakdown of macroscopic materials into individual components at the nanoscale level. Consequently, nanofibers can be produced using numerous methods, including mechanical grinding and chemical and/or enzymatic hydrolysis, to deconstruct the original structures of cellulose, hemicelluloses, proteins, and lignin.^111^ Wang et al. devised a system for obtaining CNFs by a top-down approach from a water suspension of cellulose pulp utilizing mechanical stress.^112^ The following is a quick discussion of the electrospinning technique as one of the top-down approaches in the fabrication of nanofibers.

#### 3.1.1 Electrospinning

**Figure 6:**
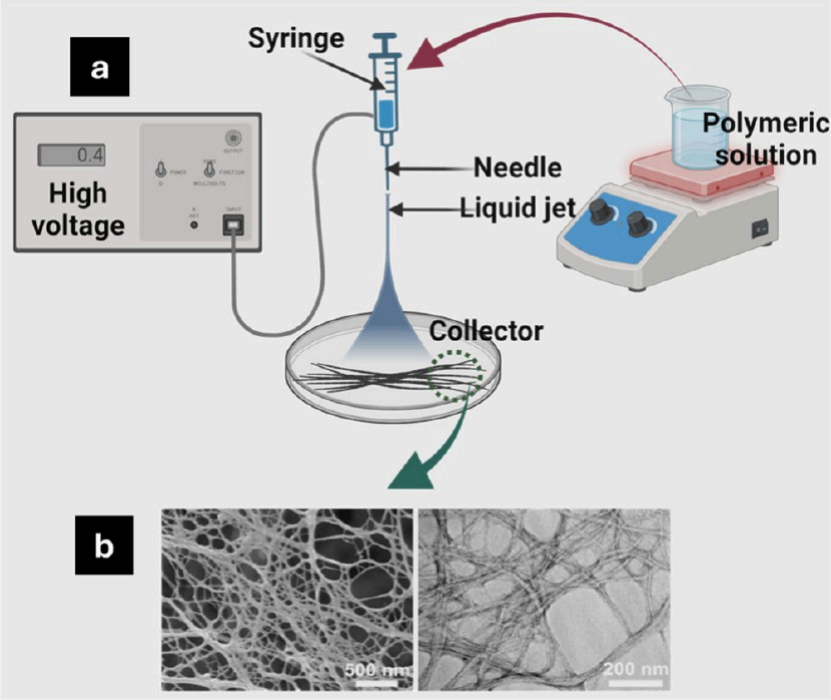
Schematic illustration for nanofiber formation via electrospinning process (a). FESEM and TEM images of cellulose nanofibers (b). Reproduced from ref (118). This is an open-access article available under the terms of the Creative Commons CC-BY license. Copyright 2021, Tuukka Nissila, Jiayuan Wei, Shiyu Geng, Anita Teleman, Kristiina Oksman.

An interesting process, which vastly uses conventional electrostatically driven technology for fabricating nanofibers with average diameters of several micrometres to nanometers, followed by considering more advantageous rather than other techniques for fabricating nanofibers.^113^ This technique comprises using a higher level of electrostatic forces; polymeric solution is drawn through the spinneret to deposit randomly on a grounded aluminum foil fibrous mat collector. An electrospinning machine is a complete package of engineered setups four prime components are associated with fabricating quality nanofibers followed by depending on the suitable solvent selection for required polymer solution preparation i.e., a syringe pump, high voltage power supply, a needle, and a collector (commonly, a metal screen, plate, or rotating mandrel).^114^ Over the last few decades, a wider variety of electrospinning techniques have been developed i.e., melt electrospinning, solution electrospinning, needleless electrospinning, coaxial electrospinning, multiple jet electrospinning, charge injection electrospinning, self-bundling electrospinning, cylindrical porous hollow tube electrospinning, nano spider electrospinning, and electro blowing.^115^ Particularly, prepared electrospun nanofiber diameter, fiber orientation with entanglement, and morphological structure have been meticulously influenced by the associated electrospinning process parameters i.e., solution flow rate, operating voltage, type of solvent used, a needle gauge, and needle tip to collector distance.^116^ Over the past few decades, there has been an increase in interest in CNFs production (Figure 6). The electrospinning technique comprises several advantages in the field of nanofiber fabrication i.e., the ability to produce thin fibers, low production cost (nanofiber production is cheaper via electrospinning rather than synthesis from natural sources), a step process for fabricating 2D or 3D nanofiber structure, enhanced substantial mechanical, physical, chemical properties produced fibers, higher aspect ratio, commercial availability, deposition of nanofiber onto various substrate with low static charges.^117^

### 3.2 Bottom-Up Approaches

A bottom-up strategy involves the aggregation of minute particles to create innovative bulk materials, such as those ranging from 1 to 10 nm in size. The physical method entails the utilization of mechanical energy or high-energy radiation to generate nanofibers. Utilizing bottom-up techniques, atoms, molecules, and nanoparticles serve as foundational elements for the construction or assembly of intricate structures and materials, commencing at the molecular scale, such as the fabrication of novel nanofiber structures through polymer synthesis.^119,120^ Berger et al. prepared self-assembled nanofiber coatings to bundle and control anti-miR-92a release to promote endothelial cell angiogenesis and overall diabetic ulcer wound healing.^121^

#### 3.2.1 Phase Separation

Another fabrication method of producing nanofibers is in which a freezing technique is included to separate a solution (i.e., hydrogel) in the solvent-rich and polymer-rich phases. According to Zahmatkeshan et al., 2019, first a polymer is dispersed into a solvent (i.e., tetrahydrofuran) followed by forming a homogeneous polymer solution.^122^ Depending on physical inconsistencies, it will be permitted to separate into two phases, with the upper polymer phase and bottom solvent phase, by either using a nonsolvent or heat treatment, resulting in gelation. In the phase separation process, the gelation of the polymer is considered the most crucial part as it is meticulously maintaining the polymer size and porous structure of the polymer.^123^ After that, association with the free-drying technique for removing solvent and moisture from the gel formed a porous nanofiber structure. Nanofibers’ structure formation may be controlled by varying the polymer concentration and temperature and utilizing different solvents. The primary processing factors regarding the phase separation process include polymer and solvent concentrations, freeze-drying temperatures, and the presence of cross-linking additives. This particular technique provides advantages such as minimal requirements of equipment, and controlled fiber pore size and structure; followed by some limitations i.e., cannot be used for all types of polymers, and a more time-consuming process in case of fabrication of the nanofibers.^124^

#### 3.2.2 Template Synthesis

Another efficient technique of fabricating nanofibers for diversified materials is termed the template-assisted synthesis short template synthesis fabrication method. Several types of nanofibers (such as those made of semiconductors, carbons, carbon nanotubes, conductive polymers, metals, metal oxides etc.) are constructed with template synthesis alone or in combination with other fabrication techniques (i.e., electrochemical deposition, chemical vapor depositions (CVD), and sol–gel method) with the template synthesis process.^125^ In template synthesis, the template can be classified into two categories; first, the hard template, which is responsible for fabricating nanorods or nanotube-like structures; second, the soft template, which is capable of producing wire-like structures followed by considering the limitations of withdrawing the template after the synthesis. Template size, shape, pore size, and nature are the process parameters included in this process.^126^

#### 3.2.3 Drawing

Drawing is an easy method for generating fibers. The primary advantage of this approach is that it necessitates only a micropipet or a sharp tip. A pointed tip is employed in this process to extract a droplet of a previously deposited polymer in the form of liquid fibers.^127,128^ The liquid is then evaporated due to the augmented surface area, resulting in the solidification of the liquid fibers. To mitigate the issue of volume shrinkage that hinders the continuous drawing of fibers and impacts their diameter, hollow glass micropipettes may be employed in place of a sharp tip with a consistent polymer dosage.^129^ The micropipette is gradually withdrawn from the liquid at a reduced velocity after being immersed in the droplet with a micromanipulator; thus, nanofibers are drawn and positioned on the surface by contacting the micropipette’s tip. This process is applied repeatedly to each droplet to produce a nanofiber.^130^ This method can be employed to produce continuous nanofibers in various configurations.^131^ Moreover, precise regulation of critical drawing parameters, including speed and viscosity, may be achieved, facilitating repeatability and control over the size of the manufactured fibers.

#### 3.2.4 Self-Assembly

Self-assembly is based on the spontaneous organization of amphiphilic chemicals that can be regarded as active molecules. As a bottom-up construction technique, self-assembly relies on the aggregation of tiny units facilitated by intermolecular forces, including hydrogen bonding, hydrophobic interactions, electrostatic processes, and biomolecule-specific interactions.^132^ These units will systematically organize and arrange to create macromolecular nanofibers. The morphology of the nanofibers is dictated by the configuration of the constituent units. This approach enables the production of nanofibers smaller than 100 nm and several micrometers in length; however, the procedure is time-intensive. The primary disadvantages of the self-assembly approach include low productivity, challenging control over fiber diameters, and a restricted selection of active compounds capable of self-assembly.^133^

## 4 Understanding Cellulose Nanofibers: From Fundamentals to Innovations

**Figure 7:**
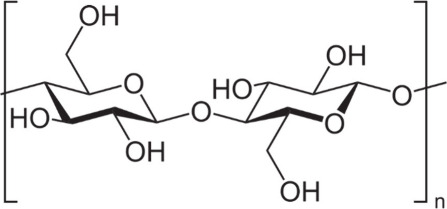
Chemical structure of cellulose. This image has been created using the creative common Biorender software (https://www.biorender.com/).

Cellulose is the most prevalent natural polymer as a renewable and biodegradable material with unique physical and chemical characteristics. Cellulose is a carbohydrate, which is represented by the formula (C_6_H_10_O_5_)n (Figure 7). It is important to note that cellulose is insoluble in aqueous solvents.

**Figure 8:**
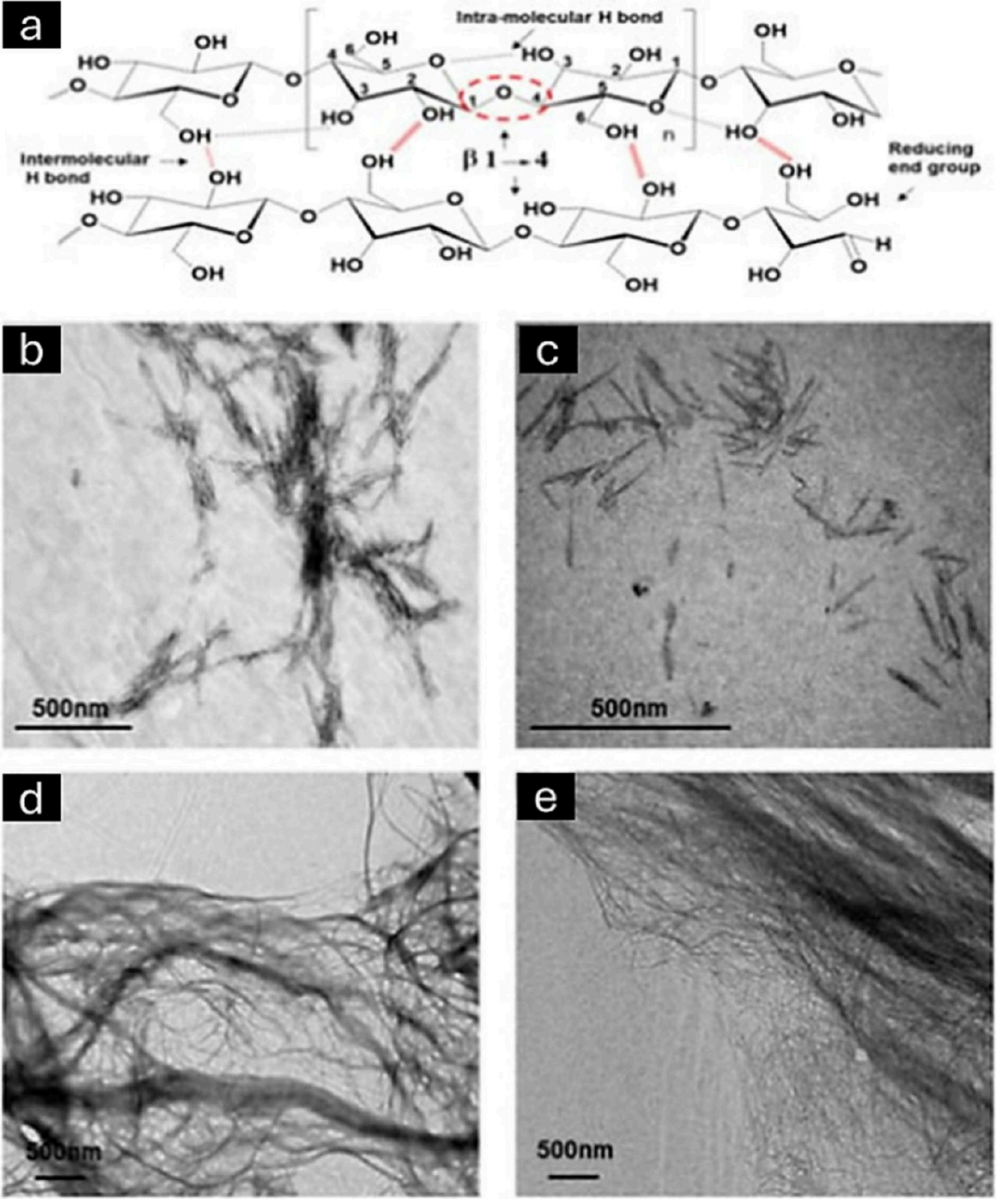
Structure of cellulose and its pattern of inter- and intrahydrogen bonding (a). Analysis of the unmodified and oxidized nanocellulose samples’ morphological and structural characteristics shown in the transmission electron micrographs are cellulose nanocrystals (b), oxidized cellulose nanocrystals (c), cellulose nanofibers (d), and oxidized cellulose nanofibers (e). Reproduced from ref (134). This is an open-access article available under the terms of the Creative Common CC-BY license. Copyright 2019, John Moohan, Sarah A. Stewart, Eduardo Espinosa, Antonio Rosal, Alejandro Rodriguez, Eneko Larraneta, Ryan F. Donnelly, Juan Dominguez-Robles.

Cellulose exhibits a tightly bound structure due to the presence of hydrogen bonds between individual chains. This results in an interstrand hydrogen bond (Figure 8a), that can readily expand and flex, allowing water to penetrate. Additionally, the high degree of polymerization of cellulose contributes to its insolubility. Figure 8b–e displays various instances of the nanocellulose structure as observed using transmission electron microscopy.

**Figure 9:**
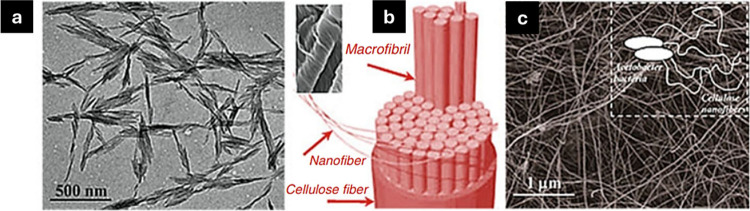
Transmission electron microscopic (TEM) images were captured for CNWs (a); nanofibers derived from cellulose fiber (b), and TEM images captured for bacterial cellulose (c). Reproduced from ref (137). This is an open-access article available under the terms of the Creative Common CC-BY license. Copyright 2014, Jun Araki, Shiho Mishima; Reproduced from ref (138). This is an open-access article available under the terms of the Creative Common CC-BY license. Copyright 2017, Luis J. Del Valle, Angelica Diaz, and Jordi Puiggali.

Presently, there is considerable interest in nanomaterials made from cellulose, because of their exceptional inherent and physical characteristics. These include a high tensile strength and elastic modulus (130–150 GPa), a large specific surface area (up to several hundreds of m^2^/g), a low density (1.6 g/cm^3^), and reactive surfaces that are also biodegradable and renewable.^135^ In recent years, several types of nanocelluloses have been divided as cellulose microfibers (CMFs), cellulose nanofibers (CNFs), cellulose nanowhiskers (CNWs) or nanocrystals (CNCs), and cellulose nanoparticles (CNPs) based on their diameter and length (Figure 9).^136^ CNFs are made by mechanically breaking down cellulose into tiny particles in a high-pressure homogenizer. CNFs are usually long and flexible, with diameters in the range of 5–60 nm and are hydrophilic due to the presence of (−OH) groups. CNFs are the most abundant, renewable, and sustainable biopolymer with exclusive properties related to stiffness, biodegradability, biocompatibility, and ability to form a strong entangled nanoporous network, with thermal and swelling behavior.

The diameter of a nanofiber (1–100 nm) is influenced by both the choice of polymer used as the base material and the method employed in its manufacturing process.^139^ For the preparation of a nanofiber, several sources of both natural and synthetic polymers are meticulously involved, i.e., cellulose, chitosan, collagen, gelatin, alginate, keratin, silk fibroin, polylactic acid (PLA), polycaprolactone (PCL), polyurethane (PU), poly(ethylene-*co*-vinyl acetate) (PEVA), and poly(lactic-*co*-glycolic acid) (PLGA).^140^ Natural polymers possess inherent bioactivity, rendering them more biocompatible and biologically potent than their synthetic counterparts.^141^ Nevertheless, their application in biomedical domains is constrained by their uneven mechanical composition. Nowadays, biomedical complex structures are constructed using a combination of natural polymers with synthetic polymers to obtain not only a high level of bioactivity and biocompatibility but also sufficient mechanical properties.^142^ Nanofibers have attracted numerous interests for use in a diversified biomedical application, i.e., wound healing, scaffold preparation, tissue engineering, cancer cell therapy, stem cell therapy, drug delivery, etc., over the last few decades because of their special functional characteristics, such as having small diameters, being lightweight, having controllable pore structure, their structural resemblance to the native cellular microenvironment, and their wider surface volume compared to high aspect ratios, which are crucial for cellular and molecular activities.^143^ Cellulose-based electrospun nanofiber fabrication is the point of interest for researchers in the case of diversified biomedical applications (i.e., wound healing and dermal implantation). Table 2 gives meticulous information about the electrospun conditions of cellulose-based nanofibers with process parameters in the field of dermal infection.

**Table 2:** Electrospun Condition of Cellulose-Based Nanofibers with Process Parameters in the Field of Dermal Infection

Cellulose Types	Electrospun condition	Critical attributes	Application	References
Carboxymethyl cellulose/Poly (ethylene oxide)	9# gauge needle (ID = 0.6 mm)	Average fiber diameter of 103 ± 30 nm	Antimicrobial dressing	(144)
	The feeding rate of the solution was 2 mL/h under a voltage of 22 kV	Nanofiber mats		
	The collecting distance between the needle tip and the collector of 10 cm	Smooth surface		
Carboxymethyl cellulose/Polyurethane	The feeding rate of the solution was 0.6 mL/h under a high voltage range of (16–20 kV)	Average fiber diameter of 255, 225.5, and 199.6 nm	Diabetic wound healing	(145)
	The distance between the tip of the needle and collector of 16 cm.			
Cellulose Acetate/Essential Oil	23-gauge needle	Average fiber diameter of 700–1500 nm	Antimicrobial wound dressings, antibiofilm surface	(146)
	Working at flow rates of 3 or 5 mL/h under a voltage of 120 kV			
	Collected fibres were placed at a distance of 15 cm from the needle			
Methylcellulose/Polyvinyl alcohol	18-gauge needle	Fiber diameter of (100–200 nm)	Carrier for Drug Delivery	(147)
	Flow rate (0.5–1) mL/h			
	Applied voltage (20–25 kV)			
	Distance (10–15 cm)			
Ethylcellulose/Hydroxypropyl Methylcellulose	21-gauge needle		Wound dressing	(148)
	Applied voltage (14–16 kV)			
	Flow rate of 1 mL/h			
	Horizontal distance of 10 cm from the needle tip			
Sodium Carboxyl Methyl Cellulose/Calcium Alginate	Flat-end metal needle (0.6 mm in inner diameter)	Average fiber diameter of 314 ± 76.45 nm	Skin tissue engineering	(149)
	High voltage DC source of 23 kV feeding at a rate of 0.8 mL/h			
	Distance of 20 cm away from the needle			
Polycaprolactone/Cellulose Acetate	21-gauge needle Constant flow rate of 1.2	The mean diameter of the fiber is 615.12 ± 139.47 nm	Wound dressings for skin regeneration	(150)
	The applied voltage of 20 kV			
	Collector distance of 15 cm	3D nanofibrous mesh was randomly oriented and nonbeaded fibres		
Cellulose Acetate	20-gauge needle	Average fiber diameter of 324 ± 94 nm	Wound dressing	(151)
	The applied voltage of 14 kV	Bead-free continuous fibers		
	The flow rate was kept at 0.5 mL/h			
	Collector to needle distance of 180 mm			
Hydroxypropyl cellulose	The applied voltage of (14–15 kV)	750–1050 nm in diameter	Tissue Engineering Fibrous mat.	(152)
	The constant flow rate of (1–2.5 mL/h)			
	Distance of 10 cm between the tip and the collector			

## 5 Application of Cellulose Nanofiber Scaffolds in Dermal Wound Treatment

Nanofiber scaffolds have recently attracted a lot of attention due to their ability to replicate the fibrous architecture of native tissue microenvironments, which is essential for homeostasis and wound healing in cutaneous ECM. For instance, fibronectin, one of the major ECM proteins in the skin, was used to create nanofiber scaffolds that aided in wound healing and skin appendage regeneration.^153^ Currently, components derived from plants are also being investigated as novel sources for nanofiber scaffold materials. It has been noted that soy protein nanofiber scaffolds hasten wound closure and lessen scarring.^154^ Electrospun nanofiber composed of different polymers or polymeric blends has shown controlled release of molecules which includes antibiotics, growth factors, proteins, silver particles, plasmids, bacteria, and viruses.^155,156^ To this end, cellulose nanofibers have been researched as a dressing material for non-healing and chronic wounds because of their significant ability to absorb fluids and create transparent layers. Furthermore, the transparent nature of cellulose nanofiber film allows for the evaluation of wound progress without the need to remove the dressing.^157^ Cellulose-based or cellulose derivatives including ethylcellulose, methylcellulose, carboxymethyl cellulose, cellulose acetate, hydroxypropyl methylcellulose, and hydroxyethyl cellulose, among others have shown their application in the management or treatment of dermal wound or infections in addition to functional agents or therapeutic molecules of interest, before and after the spinning process.^158^

**Figure 10:**
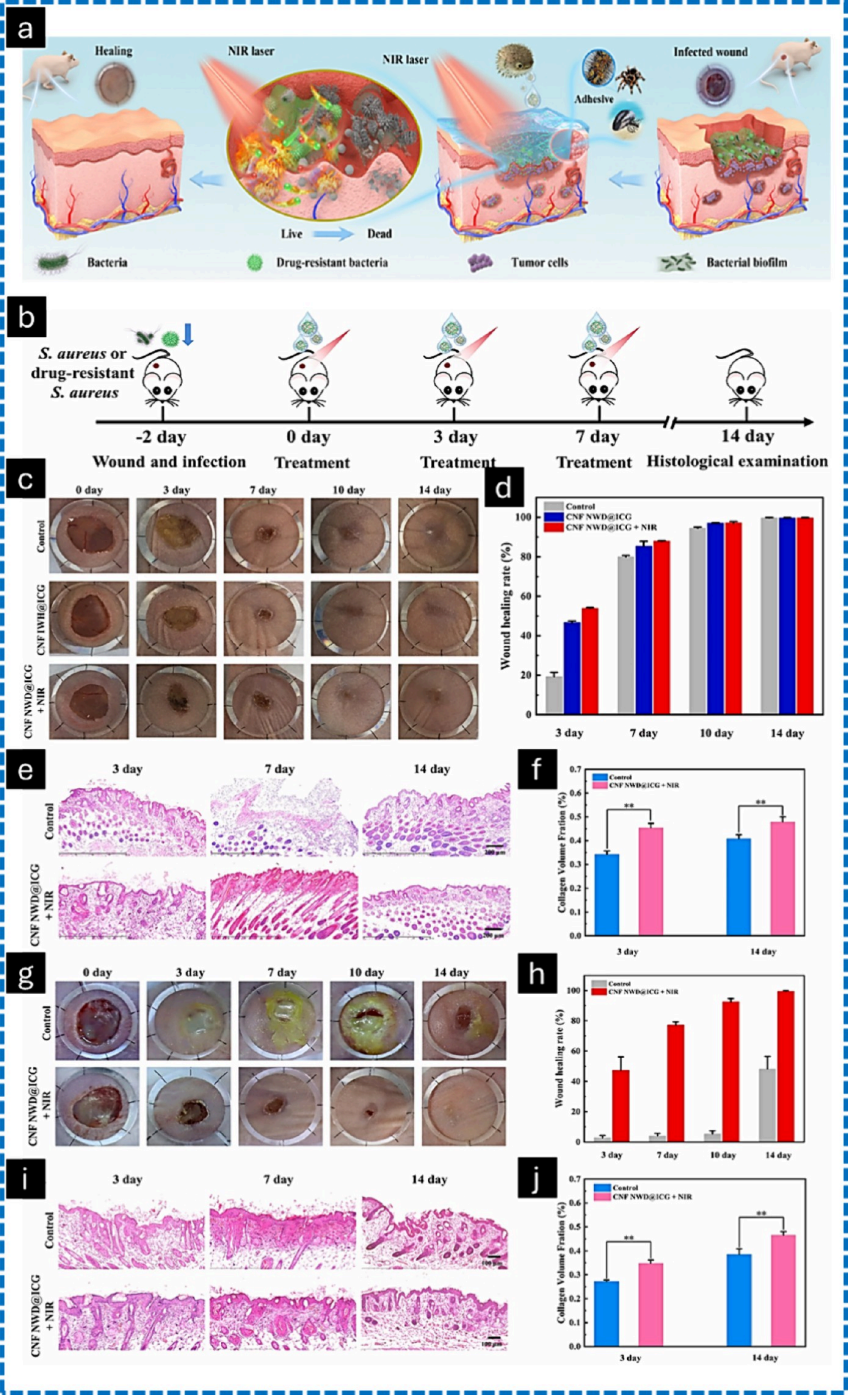
Schematic representation of CNF-based nanocage containing doxorubicin and indocyanine green and wound dressing in infected region (a). The *in vivo* wound healing efficacy of the wound dressing was assessed through an experimental procedure involving bacterial infection wounds (b); images of *S. aureus*-infected wounds in BALB/c nude mice subjected to various treatments at 0, 3, 7, 10, and 14 days (c); the associated wound healing rates (d); H&E staining (e) (scale bars: 200 μm); and the collagen volume fraction of wound tissue on days 3 and 14 (f); images of drug-resistant *S. aureus*-infected wounds in BALB/c nude mice treated with different interventions at 0, 3, 7, 10, and 14 days (g); the corresponding wound healing rates (h); H&E staining (i) (scale bars: 200 μm); and the collagen volume fraction of wound tissue on days 3 and 14 (j). Adapted with permission from ref (160). Copyright 2021, Biomaterials.

Chen et al. reported that pH-responsive and near-infrared (NIR)-responsive cellulose nanofibers containing doxorubicin (400 mg·g^–1^) and indocyanine green (25 mg·g^–1^) were prepared for photothermal therapy of tumor and infection-induced wound healing.^159^ The dual NIR-triggered wound dressing exhibited a superior photothermal conversion efficiency and demonstrated effective antibacterial activities against *Escherichia coli*, *Staphylococcus aureus*, and drug-resistant *Staphylococcus aureus*. BALB/c nude mice (6–8 weeks; female) were utilized for animal experiments to assess the inhibitory capacity of biofilm formation and the therapeutic effect on infected wounds *in vivo*. A wound model of drug-resistant bacterial infection was developed to assess the efficacy of photodynamic therapy (PDT) and photothermal therapy (PTT) of the wound dressing for the eradication of biofilms *in vivo* under NIR laser irradiation. The NIR- and pH-responsive bionic “On/Off” switches of the dressing facilitated a regulated and effective drug release onto the wound site (Figure 10). It could efficiently eradicate bacterial biofilms and exterminate the A375 tumor cells. Overall, the bionic wound dressing with shape adaptability was concluded to treat effectively irregular postoperative skin tumor wounds and drug-resistant bacterial infections through the combined therapies of photothermal, photodynamic, and chemotherapy, offering an optimal strategy for clinical intervention.

**Figure 11:**
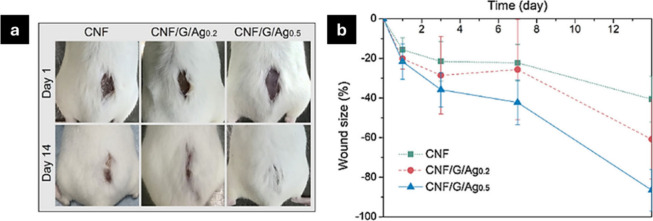
Digital photographs of the mice showing the dressing treatment with the application of silver-nanoparticle-based carboxylated cellulose nanofibers (a) and reduction in wound size with time (b). Adapted with permission from ref (163). Copyright 2018, Carbohydrates Polymers.

In another study, a green nanocomposite hydrogel was incorporated with aminated silver nanoparticles (Ag-NH_2_ NPs) and gelatin (Gel) into carboxylated cellulose nanofibers (CNFs).^161^ An interpenetrating polymeric network was established, resulting in the noncovalent cross-linked hydrogel CNF/Gel/Ag, characterized by dynamic ionic bridges. The assessment of the antibacterial efficacy of the hydrogel dressings was conducted by using two methods: optical density and disc diffusion. Quantitative studies were conducted in triplicate for *S. aureus* (ATCC 29213) and *P. aeruginosa* (ATCC 15692). Eighteen mature male Kunming mice (18–22 g) were randomly and evenly allocated into three groups to assess wound healing characteristics. On the initial day, an 8 cm thick skin wound was created on the dorsum of each mouse and securely covered with the dressing samples. The application of CNF/Gel/Ag reduced the inhibition of the two most prevalent bacteria, with CNF/G/Ag_0.5_ demonstrating superior efficacy, but treatment with CNF alone was ineffective. The study of the wound healing model, both *in vitro* and *in vivo*, for CNF/G/Ag_0.5_ demonstrated exceptional biocompatibility (about 100% viability of infected cells) and wound healing efficacy (approximately 90% healing and 83.3% survival after 14 days) (Figure 11). Taken together, the hydrogel dressing containing 0.5 mg/mL Ag-NH_2_ NPs (CNF/Gel/Ag_0.5_) exhibited enhanced mechanical strength, self-recovery, antibacterial efficacy, commendable hemostatic performance, and an optimal fluid balance on the wound bed (2093.9 g/m^2^ per day). Nanocomposite dermal drug delivery systems utilizing cellulose nanofibers grafted with titania nanoparticles, with antibacterial properties of cellulose-based nanofibers containing two model drugs (tetracycline; TC and phosphomycin; Phos), were evaluated, and they have shown potential relevance for the preparation of patch.^162^ The surface binding process between the drug molecule and modified cellulose nanofibers was examined concerning drug release and its subsequent antibacterial efficacy against common pathogens, specifically Gram-positive *Staphylococcus aureus* and Gram-negative *Escherichia coli*. The disk diffusion method and broth culture tests utilizing different quantities of model drugs included in nanocomposites were conducted to examine the antibacterial properties. The findings from the liquid broth assay further illustrated the efficacy of TC released from samples in suppressing bacterial activity. The incorporation of CNF_TiO2_TC_M1 or CNF_TC_M0, achieving a final concentration of TC in the broth at 10 mg mL^–1^, equivalent to 10 times the MIC, along with bacterial inoculation led to total growth inhibition of *S. aureus*. Identical findings were seen when the concentration of TC was reduced to 1 mg mL^–1^, specifically the MIC value for *S. aureus*. This indicated that all of the TC molecules incorporated into the cellulose nanofibers were released into the medium in a bioactive form. The antibacterial studies revealed that total growth suppression was seen for the Phos from nanocomposites with titania (CNF_TiO2_Phos_M2) sample upon immediate inoculation of *S. aureus* following the addition of citric buffer (0.03 M, pH = 6) to the growing medium. The impact of UV irradiation on the stability of the synthesized nanocomposites and their antibacterial capabilities postirradiation was examined, revealing improved stability, particularly for the TC-loaded materials. In summary, the results indicated that the synthesized nanocomposites are promising candidates for the preparation of potentially effective antimicrobial patches. The drug-loaded CNFs system was stable, and the medicinal properties of the drugs were retained even in the presence of Gram-positive bacteria.

**Figure 12:**
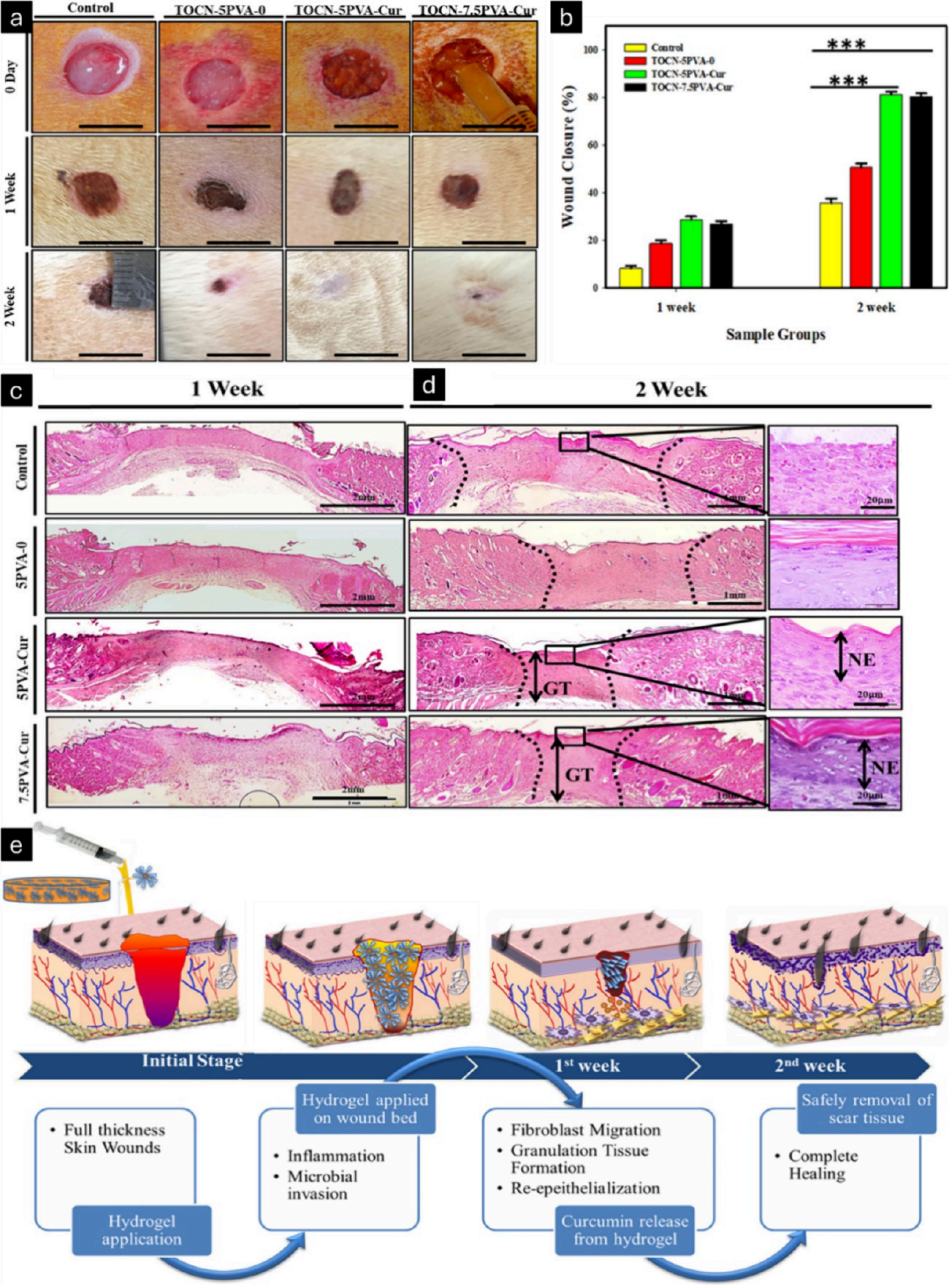
Representative images captured showing progression in wound healing after 2 weeks in *Sprague–Dawley* rats (a) and percent wound closure (b). H&E staining of skin tissue after hydrogel application after 1 week (c) and 2 weeks (d). The black dotted region illustrates the residual wound area, indicating that wound contraction is most pronounced in TOCN-5PVA and TOCN-7.5PVA-Cur. Mechanism of hydrogel in wound healing at several phases (e). The development of neo-epidermis was greater in the TOCN-5PVA-Cur and TOCN-7.5PVA-Cur hydrogel samples. **TOCN-5PVA-Cur**- TEMPO-oxidized cellulose nanofiber, with poly(vinyl) alcohol (5% w/v)-loaded with curcumin; **TOCN-7.5PVA-Cur**- TEMPO-oxidized cellulose nanofiber, with poly(vinyl) alcohol (7.5% w/v)-loaded with curcumin; **NE**- neoepidermis development; **GT**- granulation tissue. Adapted with permission from ref (164). Copyright 2019, Materials & Design.

The effect of curcumin from an oxidized cellulose nanofiber-polyvinyl hydrogel system was evaluated. This study involved the preparation of a physically cross-linked TEMPO-oxidized cellulose nanofiber, with the gelation ability of poly(vinyl) alcohol-loaded with curcumin (TOCN-PVA-Cur) hydrogel using a freeze–thaw procedure, which facilitated the release of Cur to enhance wound healing. A full-thickness excisional wound with a diameter of 10 mm was made at a location 7 ± 1 cm from the ears and tail of each rat, using a biopsy punch and forceps on the dorsum. *In vivo* studies showed an increased percentage of wound closures compared to that in the control group, after the application of oxidized cellulose-PVA curcumin nanofiber gel onto rat full-thickness skin wounds (Figure 12a,b).^164^ The dermal regeneration and re-epithelialization of wounds treated with different hydrogel compositions were analyzed using H&E staining (Figure 12c,d). Two weeks postsurgery, neoepithelium developed in the wound defects of the TOCN5PVA-Cur and TOCN-7.5PVA-Cur groups, excluding the control group. All sample groups exhibited pronounced thick granulation tissue in comparison with the control group. The TOCN-7.5PVA-Cur group had greater granulation tissue thickness compared to that of the TOCN-5PVA-Cur group. The neoepithelium was thicker in the curcumin-loaded hydrogel group compared to the control group at 2 weeks postsurgery. In summary, the fabricated TOCN-5PVA-Cur and TOCN-7.5PVA-Cur hydrogels showed efficacy in wound contraction by accelerating collagen organization.

In another study, multifunctional dermal patches made of nanocellulose and lysozyme nanofibers were prepared for cutaneous wound healing. These prepared patches were found to be biocompatible with L929 fibroblast cells. The *in vitro* wound healing assay revealed a good migration capacity representing complete wound occlusion.^165^ Bacterial cellulose (BC) nanofibers, due to their high purity, biocompatibility, and versatility, have been utilized for skin repair treatment in case of burns, wounds, and ulcers. Furthermore, BC membranes prevent infections and hasten the epithelialization process.^166^ Various studies have shown the therapeutic efficacy of benzalkonium chloride,^167^ hydroxyapatite,^168^ Aloe vera,^169^ and vaccarin,^170^ which were loaded into the BC for wound healing. Taken together, Table 3 summarizes the application of cellulose nanofibers or their derivatives in combination with therapeutic biomolecules or with other functional agents in wound healing or dermal infections.

**Table 3:** Application of Cellulose or Cellulose-Derived Nanofibers infused with biomolecules in Wound Healing

Cellulose nanofiber types	Biomolecules/functional agents	Treatment	References
Poly(vinylpyrrolidone) and ethyl cellulose	Ciprofloxacin	Wound healing	(189)
Ethylcellulose/polylactic acid/collagen	Silver sulfadiazine	Wound healing	(190)
Janus ethyl cellulose	Ciprofloxacin and silver nanoparticles	Antibacterial wound dressing	(191)
Carboxymethyl cellulose/polyvinyl alcohol	Colistin	Antimicrobial wound dressing	(192)
Ethylcellulose/hydroxypropyl methylcellulose	Aloe Vera	Wound healing	(148)
Carboxymethylcellulose/gelatin	Silk fibroin	Wound dressing	(193)
Polycaprolactone– hydroxypropyl methylcellulose	*Mentha spicata* L. essential oil	Wound infections	(194)
Carboxymethylcellulose-poly(ethylene oxide)	Vancomycin	Wound healing	(195)
Carboxymethylcellulose/polyoxyethylene	Silver nanoparticles	Wound dressing	(196)
Carboxymethylcellulose/Polyurethane	Malva sylvestris extract	Diabetic wound healing	(145)
Cellulose acetate nanofibrous patches	Chitosan/erythromycin nanoparticles	Wound and skin infections	(197)
Cellulose acetate	Modified hydroxyapatite	Wound healing	(198)
Cellulose acetate	Bioactive Sambong-oil	Skin infections	(151)
Polycaprolactone/cellulose acetate	Sorafenib	Cutaneous infections	(199)
Cellulose acetate/polyester urethane	Polyhexamethylene biguanide	Wound dressing	(200)
Cellulose acetate	Graphene oxide/TiO_2_/curcumin	Wound healing	(77)
Cellulose acetate/chitosan	Eucalyptus oil	Wound healing	(201)
Cellulose acetate/poly(ethylene oxide)	Methylene blue and ciprofloxacin	Chronic wound dressing	(202)
Cellulose acetate/chitosan hydrogel	Graphene oxide	Wound dressing	(150)
Cellulose, cellulose-chitosan, and cellulose-poly(methyl methacrylate)	Lysostaphin (Lst; cell lytic enzyme)	Wound healing	(203)
Ethylcellulose/Poly(vinylpyrrolidone)/ silk fibroin	Tetracycline hydrochloride	Wound dressing	(204)
Hydroxyethyl cellulose/polyamide 6	Tannic acid	Hemostatic wound dressing	(205)
Hydroxyethyl cellulose/polyvinyl alcohol	Collagen	Skin infections	(206)
Hydroxyethyl cellulose/chitosan/poly(ethylene oxide)	Graphene	Antibacterial wound dressing	(207)
Hydroxyethyl cellulose/β- cyclodextrin	Polyurethane	Wound dressing	(208)
Hydroxypropyl methylcellulose/Eudragit 100	Propolis	Wound dressing	(209)

**Figure 13:**
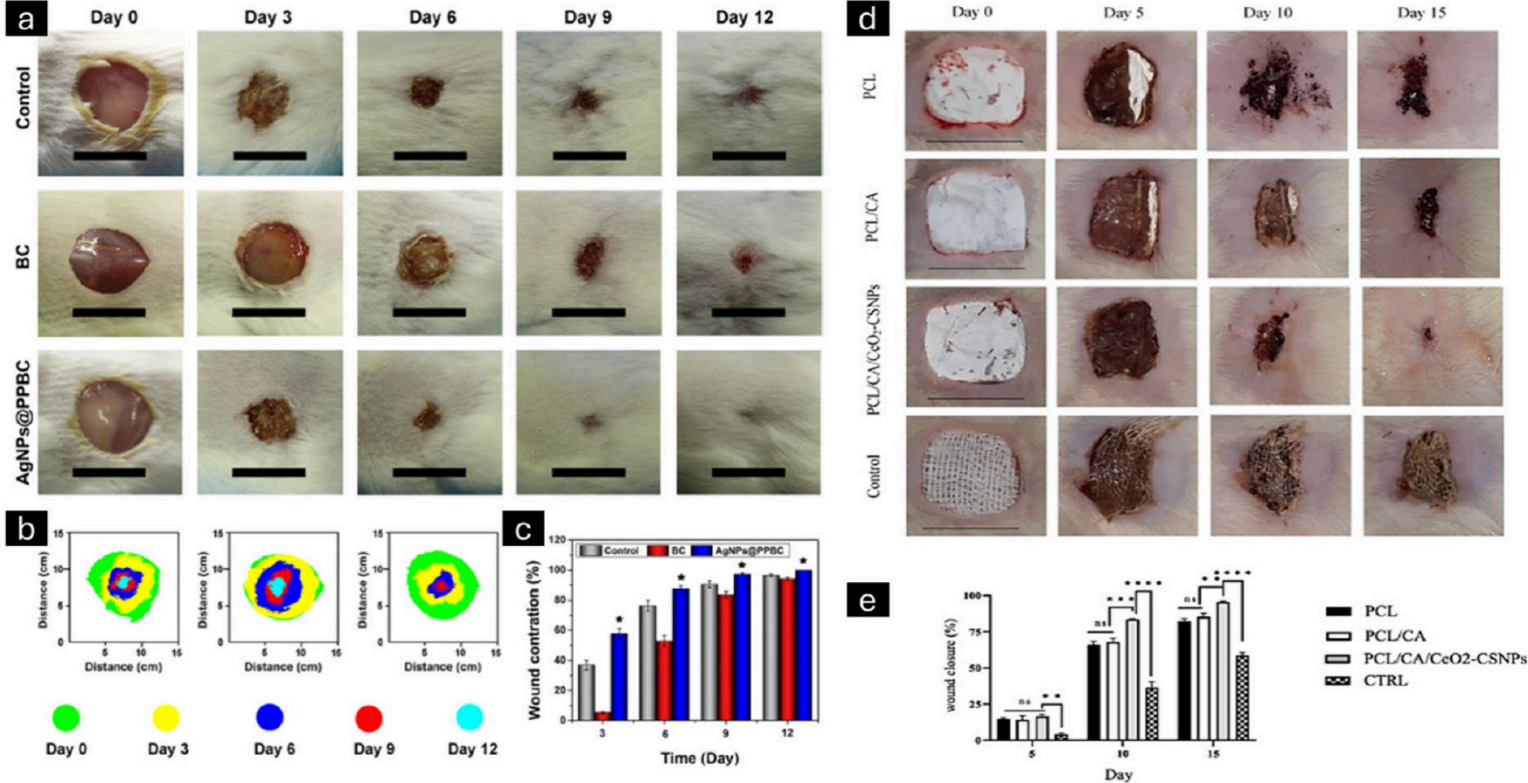
Illustrative images depicting the progression of wound healing in mice subjected to various treatments (a). The control group did not have any infection and was covered with bacterial cellulose nanofibers. The progression of wound-bed closure throughout 12 days for each treatment (b). The percentage of wound area closure in all groups showed a significant difference with the application of gold nanoparticle-based bacterial cellulose nanofibers with a p-value of less than 0.05 (c). Adapted with permission from.^171^ Digital images captured showing the ability of the fabricated PCL/CA/CeO_2_/CS) NPs nanofiber scaffolds to heal wounds *in vivo* (d) and the percentage of wound closure achieved by the nanofibers (e). Adapted with permission from ref (173). Copyright 2024, International Journal of Pharmaceutics.

In the study reported by Ma et al., dopamine (DA) was clumped in the nanofiber network structure of bacterial cellulose (BC) nanofibers.^171^ A homogeneous silver nanoparticle-loaded PDA/PEI showed sustained release of silver ions at the wound site. Furthermore, silver-nanoparticle-based BC nanofibers showed excellent antibacterial characteristics with lower cytotoxicity and improved cytocompatibility. Animal investigations showed that the fabricated material dressing at the wound site suppressed *S. aureus* infection, reduced inflammation, stimulated hair follicle development and collagen deposition, and sped wound healing (Figure 13a-c). Nontoxic, breathable, and degradable hemostatic sponges should perform rapid hemostasis with antimicrobial properties. A study by Chen et al. showed the formation of a hemostatic sponge (CQTC) with microchannels utilizing quaternized chitosan (QCS) and carboxylated cellulose nanofibers (CCNF) produced from tannic acid and Cu^2+^ complex.^172^ A liquid absorption rate test and *in vitro* hemostasis experiment showed that CQTC’s microchannels and three-dimensional porous structure improve liquid absorption and hemostasis. CQTC also enhanced wound healing and had high antibacterial activity against Gram-negative and Gram-positive bacteria. Encouraging results were shown by Kamalipooya et al. for the chitosan-encapsulated cerium oxide nanoparticles that were electro-sprayed onto electrospun nanofibers.^173^ Successful wound dressings were made using polycaprolactone/cellulose acetate/cerium oxide-chitosan (PCL/CA/CeO_2_/CS) NPs nanofiber mats. The minimum inhibitory concentration (MIC) of CeO_2_ nanoparticles, CeO_2_CS nanoparticles, and chitosan nanoparticles against *Staphylococcus aureus* was assessed using the standard broth dilution method. Samples of 100 μL were taken from all wells exhibiting no detectable bacterial growth and subsequently plated on BHI agar, which was incubated at 37 °C for 24 h. The minimum bactericidal concentration (MBC) was defined as the lowest concentration in each treated group where no bacterial colonies were present. The electrospun nanofibrous structures were evaluated for their capacity to inhibit the growth of *Staphylococcus aureus* (ATCC 25923) to determine their antibacterial efficacy. The dorsal region of the diabetic-induced rats was shaved, and a full-thickness excision wound measuring 2 × 2 cm^2^ was created following surface sterilization. The MIC test findings for *S. aureus* demonstrated the notable antibacterial efficacy of CeO_2_CSNPs at <58.59 μg/mL, whereas the MIC for CSNPs was noted at 421.87 μg/mL. Nonetheless, CeO_2_NPs exhibited a minimal antimicrobial action. Therefore, it emphasized the synergistic antibacterial activities of chitosan and CeO_2_NPs. PCL and PCL/CA nanofibers exhibited negligible antibacterial activity against *S. aureus*, whereas the mats infused with CeO_2_–CSNPs demonstrated an inhibitory zone. The research showed that the wound closure rate was enhanced with the application of PCL/CA/CeO_2_–CSNPs fiber mats in comparison to alternative scaffolds (Figure 13d, e). The observed impact was ascribed to the anti-inflammatory, antibacterial, and antioxidant capabilities of the CeO_2_NPs produced from the PCL/CA/CeO_2_–CSNPs nanofibers, which may facilitate the wound healing process. As shown by antioxidant assays and an *in vitro* wound model, the synthesized nanoparticles and nanoloaded nanofiber system had high antioxidant activity and cell migration. For up to 72 h, the hemolytic assay and cell viability study demonstrated that PCL/CA/CeO_2_–CSNPs nanofibers were biocompatible and harmless to fibroblast cells. The mats produced reduced bacterial growth significantly *in vivo*, matching the *in vitro* studies. In summary, the findings indicated that PCL/CA nanofiber mats functionalized with CeO_2_–CSNPs may be particularly successful in treating diabetes-related lesions.

**Figure 14:**
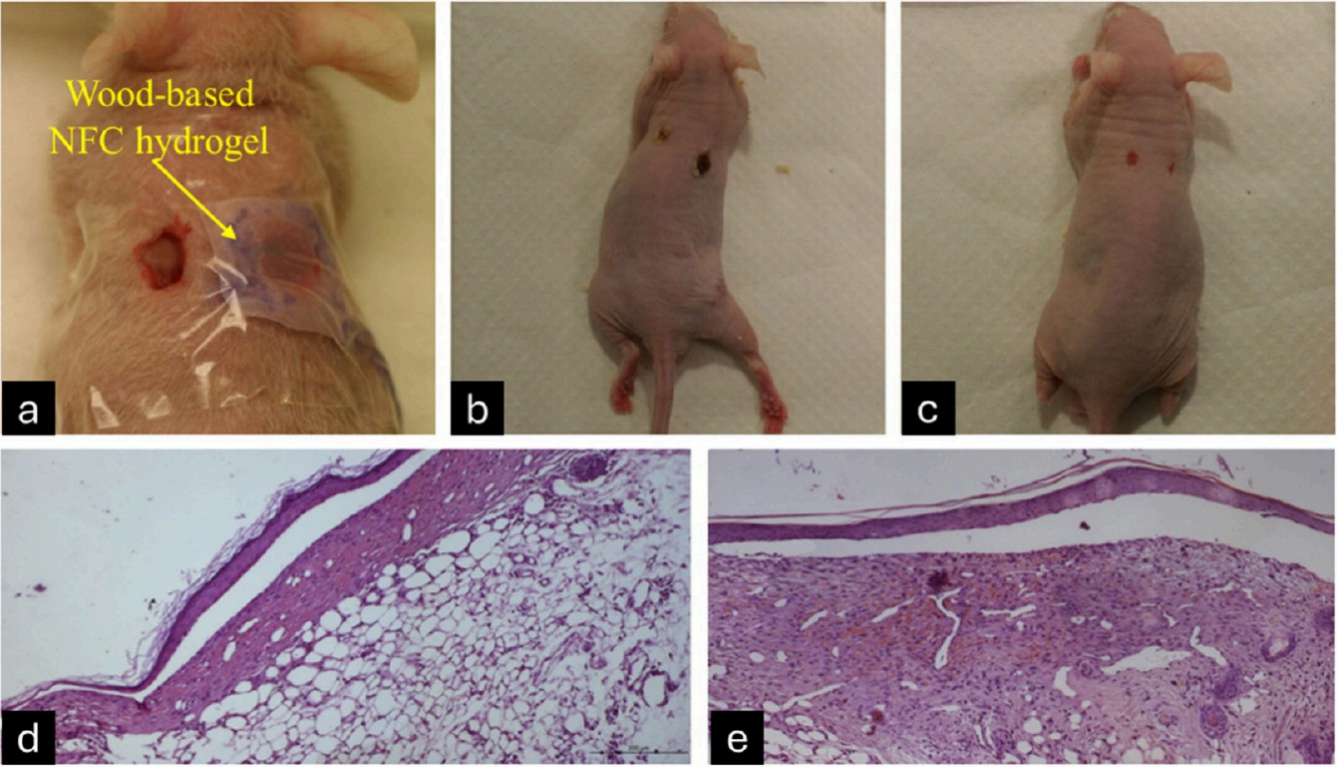
Full-thickness nude mouse model and histological study of the impact of NFC dressing on the injury site. Application of coverage and sealing to the control and treated areas following injury induction (a). Aspects of both control and treated (dressing-covered) injuries (b). Development of the region occurred on day 10. The NFC dressing covering the wound region detached from the animals after 8 to 9 days (c). Photomicrograph of the control injury stained with hematoxylin and eosin 10 days postsurgery (d). Photomicrograph of the injury treated with NFC dressing 10 days postsurgery (e). (Scale = 200 μm). Reproduced from ref (174). This is an open-access article available under the terms of the Creative Common CC-BY license. Copyright 2016, T Hakkarainen, R Koivuniemi, M Kosonen, C Escobedo-Lucea, A Sanz-Garcia, J Vuola, J Valtonen, P Tammela, A Makitie, K Luukko, M Yliperttula, H Kavola.

Hakkarainen et al. and his colleagues examined the efficacy of a wood-based NFC wound dressing in promoting wound healing.^174^ NFC dressing was assessed for antibacterial efficacy against *Staphylococcus aureus* ATCC 25923 and *Pseudomonas aeruginosa* ATCC 27853 with disc diffusion and microdilution broth techniques. No zones of growth inhibition were seen around the NFC dressing discs for these bacteria. The findings from bacterial suspension assays indicated that the inclusion of NFC dressing in the culture media did not significantly affect the growth of *S. aureus* and *P. aeruginosa* compared to the control suspension containing neat bacteria. The toxicity was evaluated for the NFC dressing upon contact with an open wound using a Swiss nu/nu mouse full-thickness skin wound healing model. Upon anesthetization, two ipsilateral injuries were inflicted on the dorsal region of each animal using a surgical punch with a diameter of 5 mm. One injury served as a control, while the other was treated with an NFC dressing. In all instances, the 100% NFC dressing became detached around days 8 to 9. No evidence of cell necrosis, or granulomas, was identified in any of the instances (Figure 14). The absence of eosinophils in the region was noted, underscoring the absence of an allergic reaction due to NFC contact. In summary, the NFC dressing was biocompatible and adhered effectively to the wound bed and separated from the wound surface following skin regeneration.

**Figure 15:**
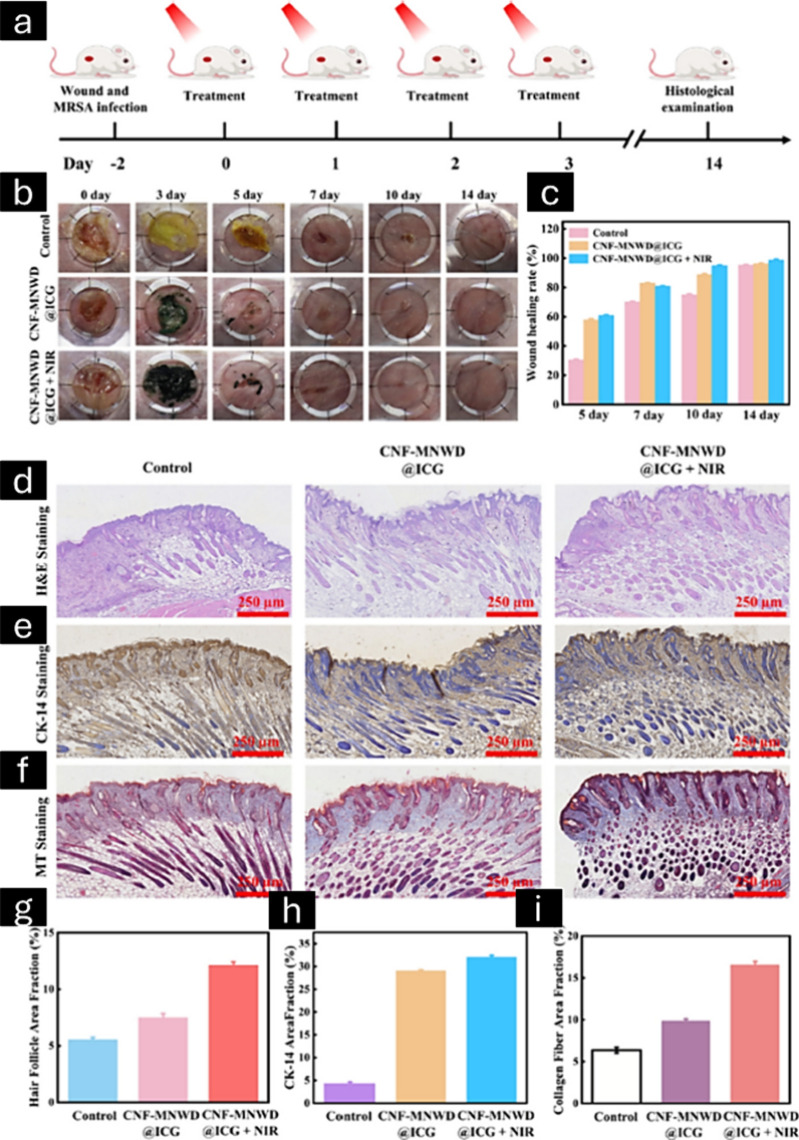
Schematic diagram of the treatment process for MRSA-infected wounds (a). Digital photographs captured of MRSA-infected wounds in mice with different treatments (b), and the corresponding wound healing rate (c). H&E staining images of the wound section on the 14th day (d) and the corresponding hair follicle area fraction (g). Images captured of cytokeratin 14 (CK-14) staining of the wound tissue on the 14th day (e) and the corresponding homologous CK-14 area fraction (h). Images captured of Masson trichrome (MT) staining of the wound tissue on the 14th day (f) and the corresponding homologous collagen fiber area fraction (i). Adapted with permission from ref.^175^ Copyright 2023, Carbohydrate Polymers.

Rapid drug loss and limited curative effects from cyclical urination contribute to wound healing difficulties following bladder tumor removal. Using pH- and near-infrared-responsive CNF nanoskeletons, magnetic responsive Fe_3_O_4_ nanoparticles, and temperature-responsive Pluronic F-127, a bioinspired CNF-based magnetic 3D nanonetwork wound dressing was fabricated for excellent tissue adhesion and biocompatibility.^175^ The dressing showed a high capacity for loading therapeutic mitomycin and indocyanine green, which formed a cohesive 3D nanonetwork at the wound site. This nanonetwork was shown to persist for an extended period and release the drug using an external magnetic field. The dressing showed strong antibacterial activity, eliminated biofilms, killed T24 cancer cells, and promoted wound healing via photothermal, photodynamic, and chemotherapeutic methods (Figure 15). Overall, this study hypothesized to have an excellent potential for bladder postoperative wound healing, thereby preventing drug loss during cyclical urination.

**Figure 16:**
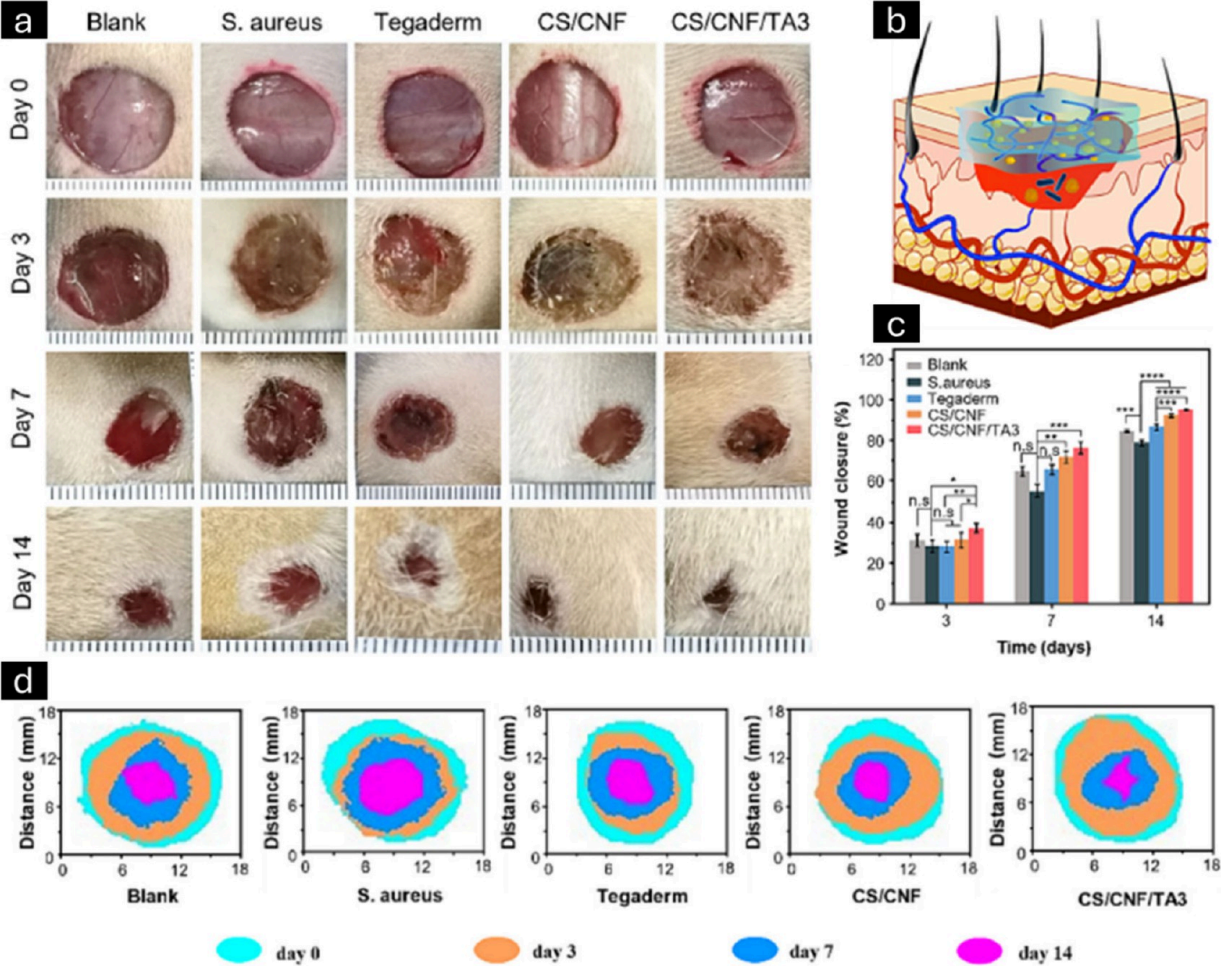
Evaluation of wound healing in infected wounds *in vivo*. Illustrative images of wounds following various treatments on days 0, 3, 7, and 14 (a). Diagram illustrating the process of treating an infected wound (b). A quantitative statistical analysis was conducted to measure the rate of wound closure at various time intervals (c). Diagram illustrating the process of closing a wound (d). Adapted with permission from ref (178). Copyright 2024, Carbohydrate Polymers.

Hydrogels derived from cellulose nanofibers are biodegradable, highly hydrophilic, and exhibit substantial mechanical strength.^176^ Hydrogels derived from cellulose nanofibers are widely employed in wound dressings and tissue engineering applications.^177^ To this end, Li et al. fabricated a novel wound dressing that effectively combats drug-resistant bacterial infections and enhances wound healing. This study involved the development of a hydrogel using electrospinning and fiber breakage-recombination techniques.^178^ The hydrogel, made of chitosan/cellulose nanofibers and tannic acid (CS/CNF/TA), demonstrated exceptional wound healing capabilities and resistance to drug-resistant bacteria. The SD rats were administered 100 μL of *S. aureus* (10^6^ CFU·mL^–1^) into their wounds for 2 h to induce infection. The inhibition rates of CS/CNF/TA against the bacterial strains exceeded 99.9%, indicating that the CS/CNF/TA hydrogel exhibited superior bactericidal efficacy. This was attributed to the incorporation of TA, a natural antibacterial, into the hydrogel that endowed it with the capacity to capture and kill microorganisms. Notably, the SEM studies confirmed that The TA damaged bacteria’s cell membrane and wall structure, causing cytoplasmic leakage and intracellular cavities that caused cells to divide into fragments. This hydrogel showed the ability to cling to the surface of tissues because it has a large number of catechol groups. In addition, it demonstrated exceptional hemostatic efficacy during the bleeding stage of the wound and thereafter maintained the wound microenvironment by absorbing water and providing moisture. Moreover, the CS/CNF/TA also stimulated the formation of blood vessels and hair follicles, speeding up the healing process of infected wound tissue. The healing rate reached over 95% for 14 days (Figure 16). Overall, the CS/CNF/TA hydrogel presented a novel method for treating drug-resistant bacterially infected wounds.

Basu et al. synthesized softwood pulp-derived cellulose nanofibers tempered with divalent calcium and copper ions to verify this method.^179^ Cross-linked hydrogels were more effective against *Pseudomonas aeruginosa*, and copper cross-linked hydrogels against *Staphylococcus epidermidis*. In addition, Ca^2+^ cross-linked cellulose nanofiber hydrogels showed the transport of drugs for chronic wound healing. Biomedical treatments can benefit from a bioadhesion system that uses the antigen–antibody interaction, where an antibody binds to a protein with a lock and key binding affinity. An involucrin antibody (SY5)-conjugated bacterial cellulose nanofiber (BCNF) coupled to involucrin (IVL) in stratum corneum corneocytes was shown to form a unique skin tissue adhesive system. SY5 was covalently integrated on the carboxylate of 2,2,6,6-tetramethylpiperidine-1-oxyl radical (TEMPO)-oxidized BCNFs via the 1-ethyl-3-(3-dimethylaminopropyl) carbodiimide (EDC)/N-hydroxysuccinimide (NHS) coupling reaction. BCNFSY5 interacted with the IVL via an antigen–antibody interaction, resulting in skin adhesion. In addition, BCNF-based skin adhesion enhanced wound healing by forming a tissue milieu for cell proliferation. These results suggested that the BCNFSY5 system could lead to a novel skin tissue regeneration adhesive.^180^

**Figure 17:**
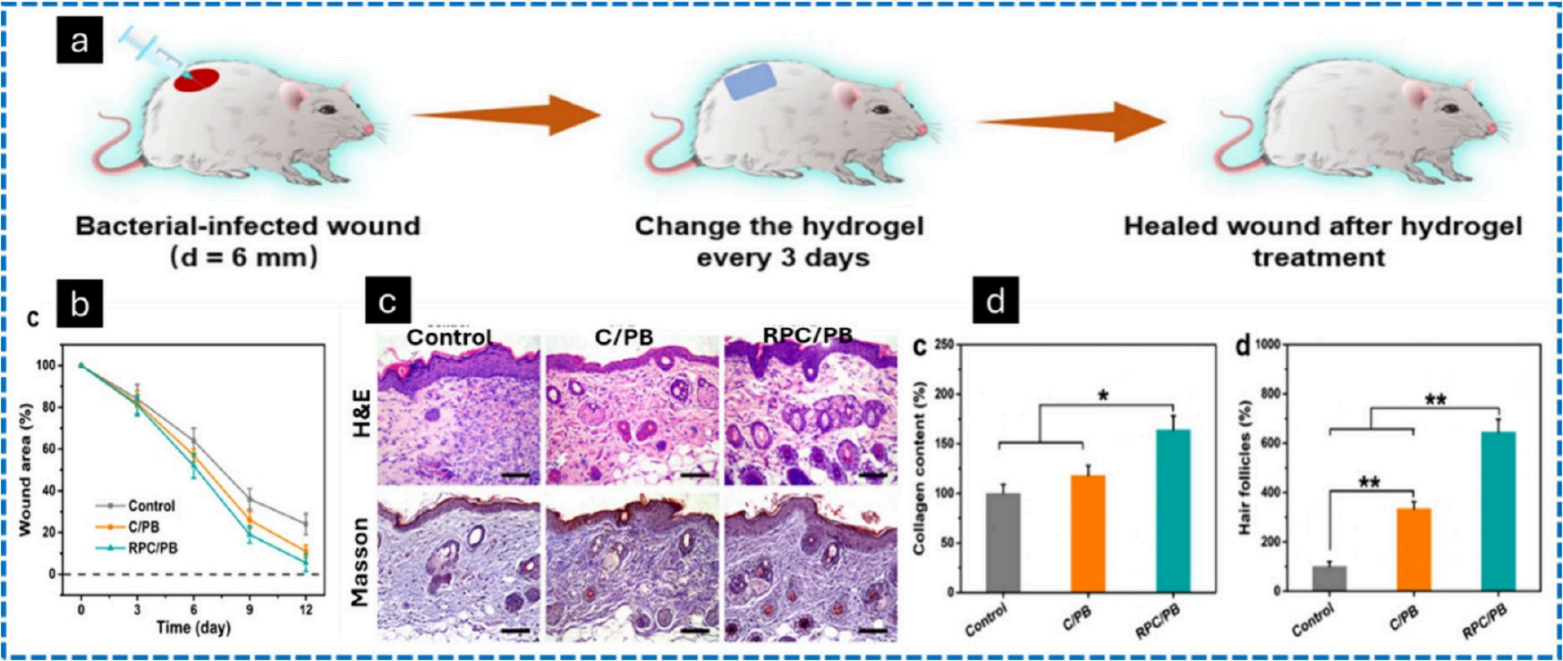
*In vivo* assessment of hydrogels in the healing of wounds infected with *S. aureus*. A schematic representation of the development of an *S. aureus*-infected murine wound model (a). Variations in wound dimensions for the control, C/PB-0.5 hydrogel, and RPC/PB-0.5 hydrogel groups (b). Micrographs of H&E-stained tissue sections and Masson’s trichrome-stained tissue sections for the control, C/PB-0.5 hydrogel, and RPC/PB-0.5 hydrogel groups following 12 days of treatment (scale bar: 200 μm) (c). Quantification of collagen levels in the control, C/PB-0.5 hydrogel, and RPC/PB-0.5 hydrogel groups by measuring hydroxyproline on day 12. The number of hair follicles in the control, C/PB-0.5 hydrogel, and RPC/PB-0.5 hydrogel groups on the 12th day, with the control group’s count designated as 100% (d). Reproduced from ref (183). This is an open-access article available under the terms of the Creative Common CC-BY license. Copyright 2022, Guihua Yang, Zhikun Zhang, Kefeng Liu, Xingxiang Ji, Pedram Fatehi and Jiachuan Chen.

Self-healing hydrogels offer notable advantages, including a minimally invasive delivery method at the target site without gel fragmentation. Self-healing hydrogels with robust tissue adhesion capabilities significantly enhance wound healing.^181^ Notably, self-healing hydrogels may offer a three-dimensional environment akin to an extracellular matrix for encapsulated cells, hence presenting the potential for tissue engineering applications.^182^ Yang et al. reported the hydrogel matrix incorporated resveratrol-grafted cellulose nanofibrils (RPC) conjugate within the polyvinyl-borax (PB) network, resulting in a semi-interpenetrating network that demonstrated substantial mechanical properties (fracture strength of 149.6 kPa), elevated self-healing efficiency (>90%), and superior adhesion performance (tissue shear stress of 54.2 kPa).^183^ The induced RPC/PB hydrogel exhibited pH-responsive drug release characteristics, with the cumulative release of resveratrol at pH 5.4 being 2.33 times greater than that at pH 7.4, thereby aligning effectively with the acidic wound microenvironment. This RPC/PB hydrogel demonstrated superior biocompatibility and antioxidant properties. Furthermore, both *in vitro* and *in vivo* findings demonstrated that the RPC/PB hydrogel exhibited exceptional antibacterial properties, facilitated skin tissue regeneration, and enhanced wound closure efficacy (Figure 17). In a different set of experiments, a novel injectable antibacterial chitosan hydrogel, exhibiting excellent self-healing capabilities and enhanced mechanical properties, has been developed using dialdehyde bacterial cellulose (DABC) nanofibers as a nontoxic biocrosslinker.^184^ The CS-DABC-5 hydrogel served as the representative for assessing the antibacterial activity against *E. coli* and *S. aureus*. In comparison to the control, the colony count was markedly reduced, indicating that the CS-DABC-5 hydrogel had a significant antibacterial action and therefore can be used as an ideal wound dressing.

Taken together, cellulose nanofibers are attractive biomaterials for pharmaceutical and biomedical applications due to their mechanical properties, highly reactive surface, biocompatibility, and biodegradability. Encouraged by the results from the laboratories, a couple of clinical trial investigations have been reported.^185,186^ Notably, in recent times, the innovative 3D printing approach has emerged as a potential means of ensuring the scaling up of the manufacture of cellulose nanofibers-based hydrogels for wound healing.^187,188^

## 6 Challenges and Prospects

The therapeutic activity of the nanofibers can be significantly enhanced by entrapping active molecules that need to be targeted at the site of action. Electrospinning technology is an old method that has been used to produce drug-loaded nanofibers due to its simplicity and potential to be compatible with other methods.^210^ Despite the increasing interest observed in the field of electrospinning in the past decade, the efficacy of electrospun nanofibers has faced a major challenge in creating comparable scaffolds due to the various parameters that influence the characteristics of the fibers. To this end, 3D printing, or the combination of 3D printing and electrospinning, has been shown to achieve biomimetic production of nanofibers without any significant variations to increase its utility in the field of wound healing. Also, the approach of interpenetrating polymeric network (IPN), can be implemented further for the fabrication of layer-by-layer assembly of nanofibers, which might help control the degradation rates for the cellulose nanofibers containing peptide drugs.^211,212^ The fields of tissue engineering and regenerative medicine are poised to revolutionize the future of skin regeneration and wound healing. To attain an optimal skin substitute, it is imperative to consider various attributes, including enhanced vascularization facilitated by bioreactors to promote vessel development, a prolonged lifespan, and seamless integration with the host tissue. Ideally, scaffold polymers, growth factors, and all cell lines should closely resemble the natural structure and function of the skin. To achieve this objective, it is necessary to incorporate additional cellular components, such as melanocytes and hair follicles, into the three-dimensionally constructed scaffolds. To this end, microfluidic skin printing and automated tissue printing are novel methodologies that have the potential to significantly transform tissue engineering approaches.^213^

Based on the discussion so far, there has been a notable surge in the utilization of cellulose nanofibers with a particular emphasis on their application in the field of wound healing. To facilitate the process of wound healing, cellulose polymers must possess notable antibacterial activity. The investigation of antimicrobial activity in nanocellulose-based materials represents a novel and complex approach. The scientific investigations have demonstrated the significance of modifying nanocellulose material in the context of its application for wound healing. In recent years, numerous studies have shown significant advances in the production of nanocellulose in pilot plants. However, the overall development of cellulose nanofibers on a larger scale has not been accomplished, despite the cost-effectiveness of the cellulose polymer.^214^ Additionally, certain obstacles impede the efficient and economic utilization of antibacterial nanocellulose-based materials. These challenges encompass the absence of suitable techniques for fabricating wound dressing films, hydrocolloid dressings, foam dressings, and packaging materials intended for wound dressing purposes. Therefore, the challenge of producing large-scale customized cellulose-based nanofibers at a low cost and with minimal energy remains a significant issue. The current research regarding the modification of nanocellulose for wound healing, specifically focusing on its antibacterial characteristics and environmentally friendly approach, remains limited and lacks comprehensive exploration. The perceptions, thoughts, or ideas to mitigate these challenges are hypothetical and therefore require extensive studies or proof-of-concept.

## 7 Conclusion

Topical drug delivery is a promising method for achieving localized therapeutic benefits in the treatment of skin infections or wounds, therefore mitigating the potential adverse effects on the entire body. The field of transdermal drug administration presents significant potential for enhancing systemic drug delivery by addressing the challenges including low oral bioavailability and the discomfort and inconvenience of injections. To this end, topical or transdermal application of nanofibers has shown exceptional performance as wound dressing materials by improving patient compliance. Nanofibers possess notable advantages compared to traditional dosage forms such as films, sponges, hydrogels, etc., which include their elevated surface area to volume ratio and considerable porosity and flexibility. Moreover, the drug release pattern of the encapsulated drugs from the nanofibers can be modulated based on the diameter of the nanofibers or alteration of the morphology to a core–shell configuration. Electrospun nanofibers have emerged as the best technology that enables the mass manufacturing of nanofibers in a straightforward and scalable manner. However, the translational approaches of nanofibers from laboratory-scale experimentation to practical utilization are hindered by the challenges associated with scaling up the electrospinning process. These challenges include the need to effectively control the pore size and ensure consistent diameters of the nanofibers. Functional polymeric nanofibrous biomaterials play a crucial role as a class of artificial extracellular matrix. Moreover, natural and synthetic polymeric biomaterials with their inherent and diverse properties have been merged with nanofibers for the development of skin substitutes or grafts, which are also extensively used as commercial products. However, the implementation of artificial skins in clinical settings necessitates the utilization of predictive testing procedures and the establishment of suitable standards and regulations. These measures are crucial to guarantee consistent replication and functional dependability of the grafts. Taken together, biomaterial-based polymeric drug-loaded nanofibers that replicate the micro- and macro-structures of various native extracellular matrices exhibit enhanced relevance and functionality, making them attractive for the treatment of skin infections and biomedical applications.
